# Therapeutic hypothermia in newborns: evidence-based guidelines from a systematic review

**DOI:** 10.1186/s13052-026-02266-x

**Published:** 2026-04-27

**Authors:** Gina Ancora, Francesca Gallini, Monica Fumagalli, Gilda Cassano, Vittoria Paoletti, Massimiliano De Vivo, Gianluca Visintin, Domenico Marco Romeo, Lucrezia De Cosmo, Isotta Guidotti, Katia Rossi, Ida Sirgiovanni, Fabrizio Ferrari, Alessandro Scoppa, Maria Vendemmia, Luca Bedetti, Licia Lugli, Luca Ramenghi, Massimo Agosti, Luigi Orfeo, Fabio Mosca, Fabio Facchinetti, Roberto Bellù, Irene Cetin, Enrico Cocchi

**Affiliations:** 1 Neonatal Intensive Care Unit, AUSL Romagna, Infermi Hospital, Rimini, Italy; 2 Neonatal Intensive Care Unit, Ospedale Isola Tiberina - Gemelli Isola, Università Cattolica del Sacro Cuore, Rome, Italy; 3 Neonatal Intensive Care Unit, Fondazione IRCCS Ca’ Granda Ospedale Maggiore Policlinico, Milan, Italy; 4 Department of Clinical Sciences and Community Health, University of Milan, Milan, Italy; 5 Neonatal Intensive Care Unit, IRCCS AOUBO, Bologna, Italy; 6 Neonatology and Neonatal Intensive Care Unit A.O.R.N Monaldi AO dei Colli, Napoli, Italy; 7 Neonatal Intensive Care Unit, hospital of Treviso, Treviso, Italy; 8 Pediatric Neurology, Fondazione Policlinico A. Gemelli, IRCCS, Rome, Italy; 9 Neonatology and Neonatal Intensive Care Unit, POC SS. Annunziata Taranto, Taranto, Italy; 10 Neonatology and Neonatal Intensive Care Unit, University-Hospital of Modena, Modena, Italy; 11 Department of Biomedical Sciences, Humanitas University, Italy; Neonatology and Neonatal Pathology Unit, Humanitas Hospital, Milan, Italy; 12 Neonatology and Neonatal Intensive Care Unit, Ospedale del Mare Asl Napoli 1 Centro, Napoli, Italy; 13 Department of Translational Medical Sciences, Division of Neonatology, University of Naples Federico II, Napoli, Italy; 14 Neonatal Intensive Care Unit, IRCCS Giannina Gaslini Institute, Genoa, Italy; 15 Neonatal Intensive Care Unit, Filippo Del Ponte Hospital, ASST Settelaghi, Varese, Italy; 16 Department of Medicine and Surgery, University of Insubria, Varese, Italy; 17 Obstetrics and Gynecology Unit, Department of Medical and Surgical Sciences, University of Modena and Reggio Emilia, Modena, Italy; 18 Neonatal Intensive Care Unit, Manzoni Hospital, Lecco, Italy; 19 Obstetrics Unit, Fondazione IRCCS Ca’ Granda Ospedale Maggiore Policlinico, Milan, Italy; 20 Department of Medical and Surgical Sciences, Alma Mater Studiorum - University of Bologna, Bologna, Italy; 21 Neonatal and Pediatric Intensive Care Unit, AUSL Romagna, Ravenna, Italy; 22 Department of Medical and Surgical Sciences (DIMEC), University of Bologna, Via G. Massarenti, 9, 40126 Bologna, Italy

**Keywords:** Newborn, Asphyxia, Hypoxic-ischemic encephalopathy, Therapeutic hypothermia, Neurodevelopment, Newborn care, Guidelines

## Abstract

Hypoxic-ischemic encephalopathy represents a major cause of neonatal mortality and long-term neurodevelopmental impairment. Therapeutic hypothermia has become the standard of care, significantly improving survival and outcomes. Nevertheless, its clinical implementation often lacks a standardized operational framework to ensure consistent efficacy and safety. The present work aimed to develop GRADE-based operational guidelines for therapeutic hypothermia, offering neonatologists a structured approach for identifying, treating, and monitoring newborns throughout the procedure. A panel of experts appointed by the Italian Society of Neonatology conducted a systematic review (PROSPERO registration CRD420250651303) of randomized controlled trials evaluating the effects of therapeutic hypothermia on neurodevelopmental outcomes. Literature searches were performed across Medline, Cochrane, Scopus, Embase, and Web of Knowledge databases, and studies were assessed using the Cochrane Risk-of-Bias 2 tool. Recommendations were formulated through consensus meetings following GRADE methodology and subsequently validated by an external multidisciplinary panel. Eight randomized controlled trials, encompassing a total of 1,843 newborns, met the inclusion criteria. Based on a comprehensive evaluation of study indications, characteristics, and outcomes, evidence-based recommendations were established. The risk-of-bias assessment revealed an even distribution between studies categorized as low and moderate risk. The resulting guidelines present a structured protocol covering all phases of therapeutic hypothermia - timing, clinical setting, management, and follow-up - and include proposals for expanding its application. To facilitate clinical implementation, the guidelines are accompanied by summary tables, graphical flowcharts, and algorithms designed for immediate use in neonatal intensive care settings. The GRADE-based recommendations presented here, now adopted as the Italian national clinical guidelines for therapeutic hypothermia in newborns, provide an evidence-driven and standardized framework to optimize neonatal care. These guidelines are intended to support daily clinical decision-making and will undergo periodic updates to incorporate emerging evidence and maintain their clinical relevance.

**Supplementary Information:**

The online version contains supplementary material available at 10.1186/s13052-026-02266-x.

## Introduction

### Hypoxic-ischemic encephalopathy in neonates

Hypoxic-ischemic encephalopathy is a severe neurological disorder resulting from inadequate oxygen and blood supply to the brain during the perinatal period [1]. It is a leading cause of neonatal mortality and long-term neurodevelopmental impairment, affecting up to 1.5 per 1,000 live births in high-income countries [2, 3]. The mortality rate among untreated infants ranged from 36% to 57%, with 31–42% of survivors developing cerebral palsy [4]. Postnatal hypoxic-ischemic events, such as Sudden Unexpected Postnatal Collapse (SUPC), primarily caused by acute airway obstruction, can also lead to encephalopathy [5, 6].

### Pathogenetic mechanisms

Brain injury in hypoxic-ischemic encephalopathy progresses through four distinct phases, extending from the initial insult to months post-reperfusion [7–9].

- *Acute Phase*: occurs during the hypoxic event, leading to neuronal necrosis and cellular damage.
- *Latent Phase*: a brief period of metabolic recovery following re-oxygenation, though cellular injury continues.
- *Secondary Phase*: begins ~6 hours post-event, characterized by metabolic failure, inflammation, and apoptosis, often clinically marked by seizures.
- *Tertiary Phase*: extends weeks to months, featuring chronic inflammation and impaired neurodevelopment, contributing to long-term deficits.

**Fig. 1:**
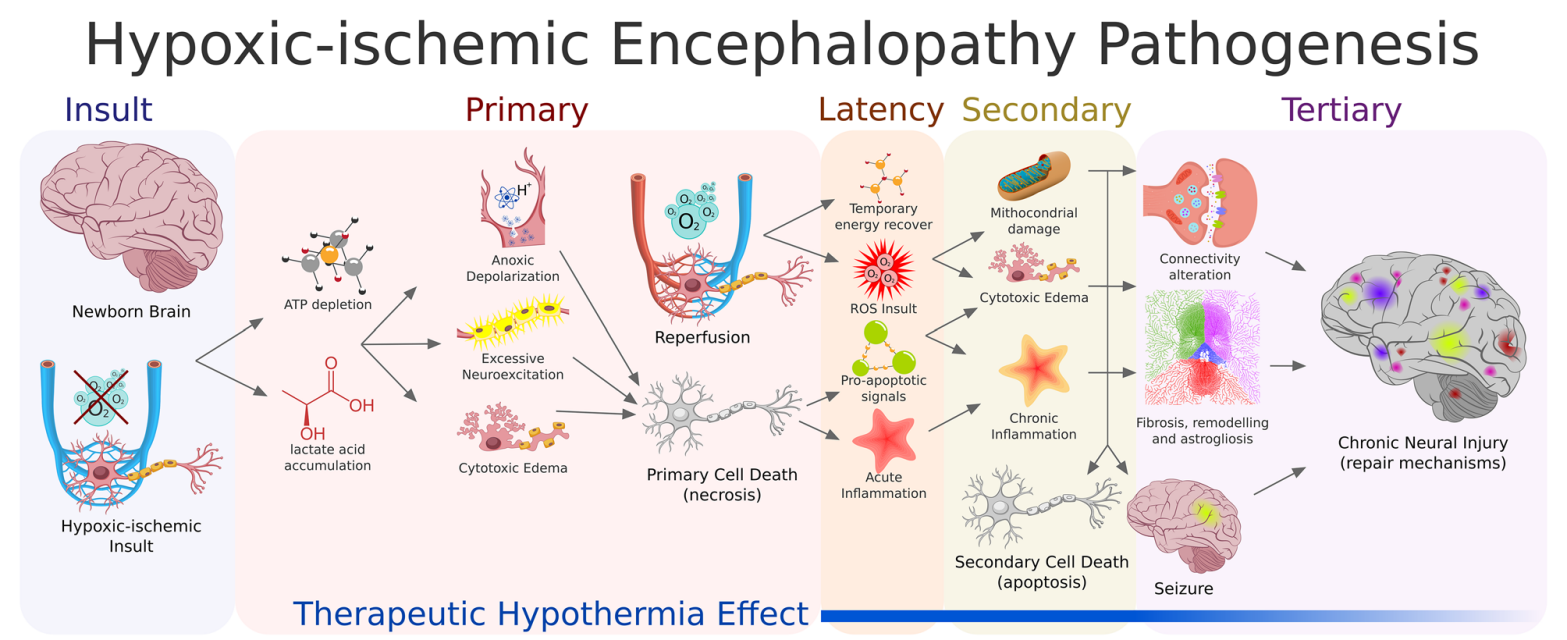
Hypoxic-ischemic encephalopathy pathogenesis and the effects of therapeutic hypothermia. This figure illustrates the pathophysiological progression of hypoxic-ischemic encephalopathy across four phases: insult, primary, latency, secondary, and tertiary. The primary phase involves ATP depletion, anoxic depolarization, excessive neuroexcitation, and cytotoxic edema. The latency phase provides a brief window of metabolic recovery before the secondary phase triggers oxidative stress, inflammation, mitochondrial damage, and apoptosis. The tertiary phase results in chronic neural injury, fibrosis, connectivity alterations, and seizures. The neuroprotective effects of therapeutic hypothermia are emphasized at the bottom, highlighting its role in mitigating neuronal injury and preserving brain function and development

A detailed timeline and pathophysiological mechanisms are illustrated in Fig. 1.

### Therapeutic hypothermia

Therapeutic hypothermia is the standard of care for moderate to severe hypoxic-ischemic encephalopathy, significantly reducing mortality and long-term disability when initiated within the first six hours of life. Numerous randomized controlled trials (RCTs) and meta-analyses confirm its efficacy [10–20]. The neuroprotective effects include [18, 21–23]:

- Reduction in vasogenic edema
- Suppression of excitotoxic neurotransmitter release
- Mitigation of oxidative stress and cytokine activation
- Lowering cerebral metabolism

### Study aim

This study aimed to develop operational guidelines for therapeutic hypothermia in high-income countries, offering neonatologists a structured framework to promptly identify and manage eligible newborns. We systematically reviewed the literature on therapeutic hypothermia, focusing on inclusion criteria and its impact on neurodevelopment, to develop comprehensive, evidence-based clinical guidelines covering its clinical indications, applicability, procedural steps, and implementation setting, throughout treatment. Developed by a panel of experts using the Grading of Recommendations Assessment, Development and Evaluation (GRADE) methodology [24], these guidelines are intended to offer detailed, evidence-based recommendations aimed at optimizing the safe and effective application of therapeutic hypothermia in clinical settings.

## Methods

### Systematic review and guidelines Development

**Fig. 2:**
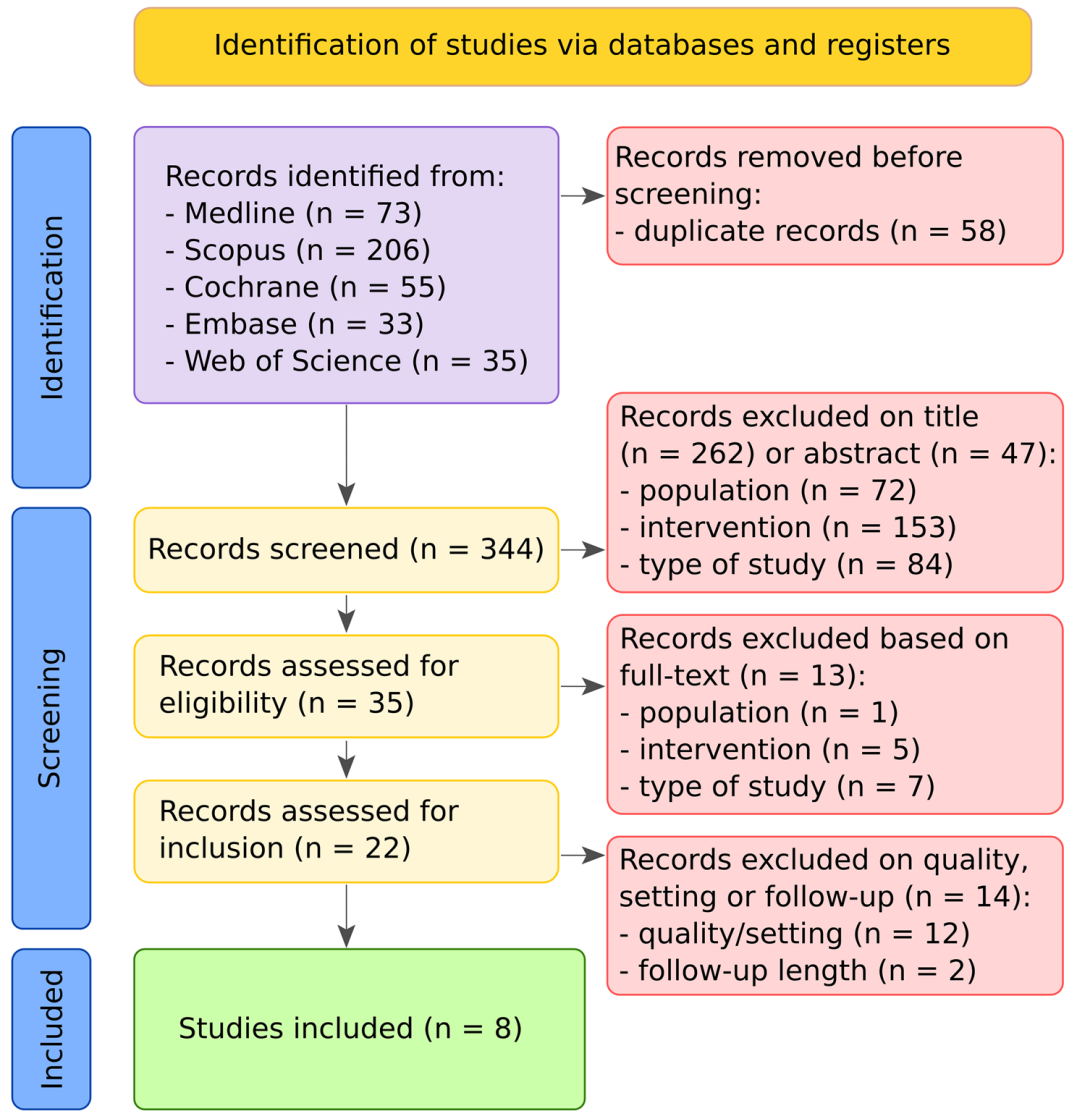
PRISMA flow diagram for study selection. A systematic review process visualized using the PRISMA framework, detailing identification, screening, eligibility assessment, and inclusion of studies. Final inclusion required neurodevelopmental assessment at 18–24 months and an overall risk of bias 2 (RoB2) judgment of low risk or some concerns (no high risk). A total of 344 records were screened after removing duplicates, with 8 studies included in the final analysis. Reasons for exclusion at different stages (e.g., population mismatch, intervention differences, study type) are depicted

**Fig. 3:**
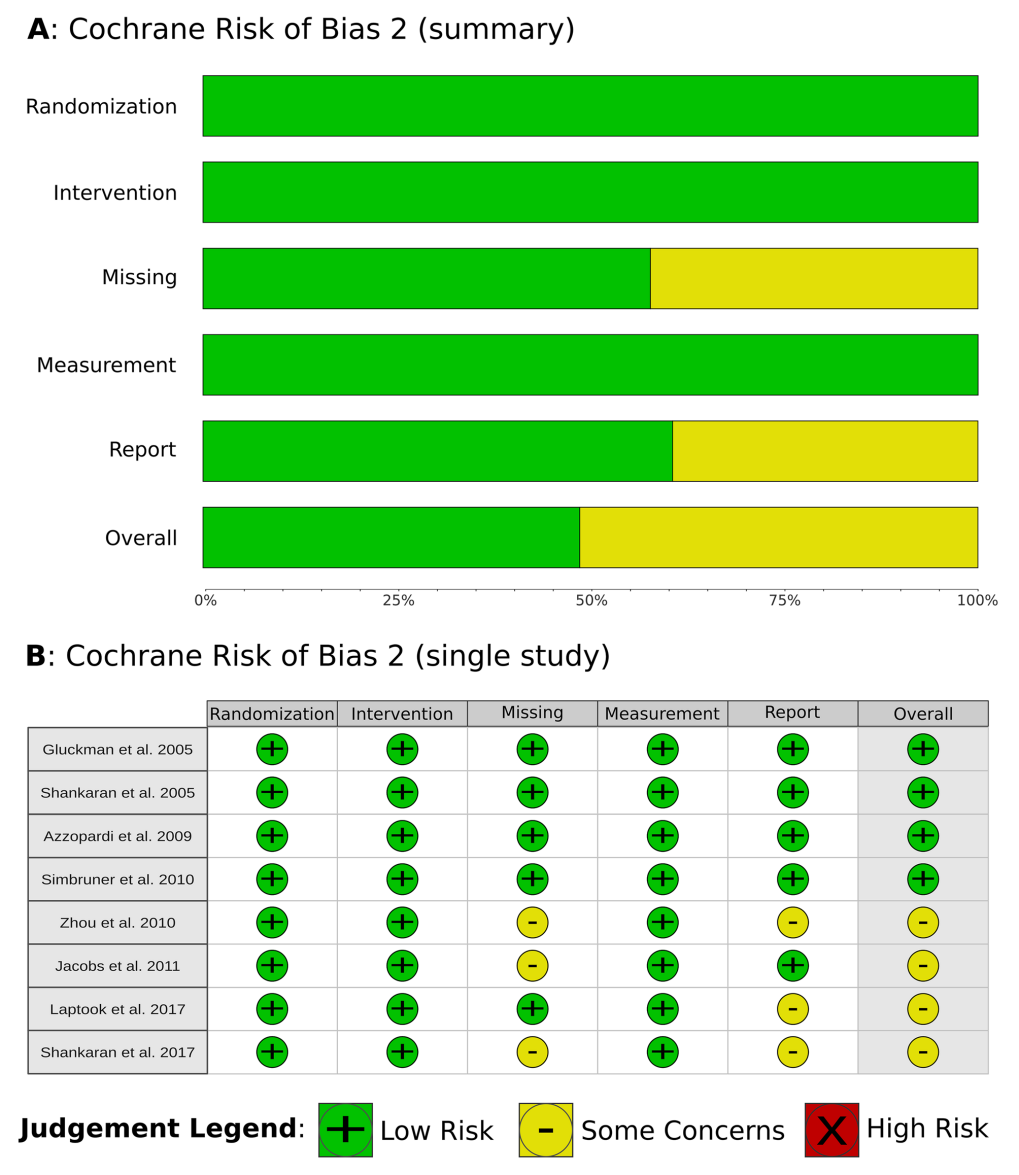
Cochrane risk of bias 2 (RoB2) assessment for included studies. Panel a presents a weighted summary of bias risk across five domains (randomization, intervention, missing data, outcome measurement, and reporting). Studies exhibit low to moderate risk, with some concerns in missing outcome data and reporting bias. Panel B provides an unweighted detailed risk of bias assessment per single study, categorizing them as low risk (+, green), some concerns (-, yellow), or high risk (x, red). The overall risk assessment informs confidence in the evidence synthesized

A multidisciplinary panel of neonatologists, pediatric anesthesiologists, and neuropsychiatrists, all experts in hypoxic-ischemic encephalopathy and therapeutic hypothermia, was commissioned by the Italian Society of Neonatology to update the 2012 second edition of these guidelines using the GRADE methodology [25]. The resulting third edition of the guidelines was published online in November 2023 by the Italian Society of Neonatology [26]. A systematic literature review was conducted in accordance with PRISMA guidelines, searching Medline, Scopus, Cochrane, Embase, and Web of Science (PROSPERO protocol number: CRD420250651303). The detailed search strategy is presented in Supplementary Fig. 1. Only RCTs meeting predefined eligibility criteria and evaluating neurodevelopment at 18–24 months were included. Study quality was assessed using the Cochrane Risk of Bias 2 (RoB2) tool and only trials judged low risk or some concerns were included, while high risk trials were excluded. Exact details on search strategy, selection criteria, data extraction, and quality assessment are provided in the supplementary methods. The PRISMA flowchart (Fig. 2) and the RoB 2 summary (Fig. 3) illustrate the study selection process and quality assessment results. Each working group member was assigned specific outcomes, critically reviewing and evaluating the literature using the GRADE system. Recommendations were finalized through monthly consensus meetings over a year, incorporating insights from the European Standards of Care for Newborn Health [27]. The document underwent critical review by external multidisciplinary experts and received approval from multiple professional societies.

### GRADE methodology

The GRADE methodology categorizes evidence quality based on study type and the likelihood that further research will change effect estimates. It supports clinical decision-making by categorizing the reliability of available evidence in:

- *High-quality*: further research is unlikely to change conclusions.
- *Moderate-quality*: future studies may slightly affect estimates.
- *Low-quality*: further research is likely to alter effect estimates.
- *Very low-quality*: significant uncertainty, high potential for future research to alter conclusions.

Overall recommendations are formulated based on quality of evidence, risk-benefit analysis, patient values, and resource considerations. The resulting classification includes:

- *Strong*: benefits clearly outweigh risks, supporting universal application.
- *Weak/Conditional*: uncertainty remains, requiring an individualized approach.
- *Research context*: insufficient evidence (equipoise); intervention may be considered within a research setting, with emphasis on informed consent and ethical considerations.

Further methodological details are available in the Extended Methods section of Supplementary Materials.

### Guidelines organization

**Fig. 4:**
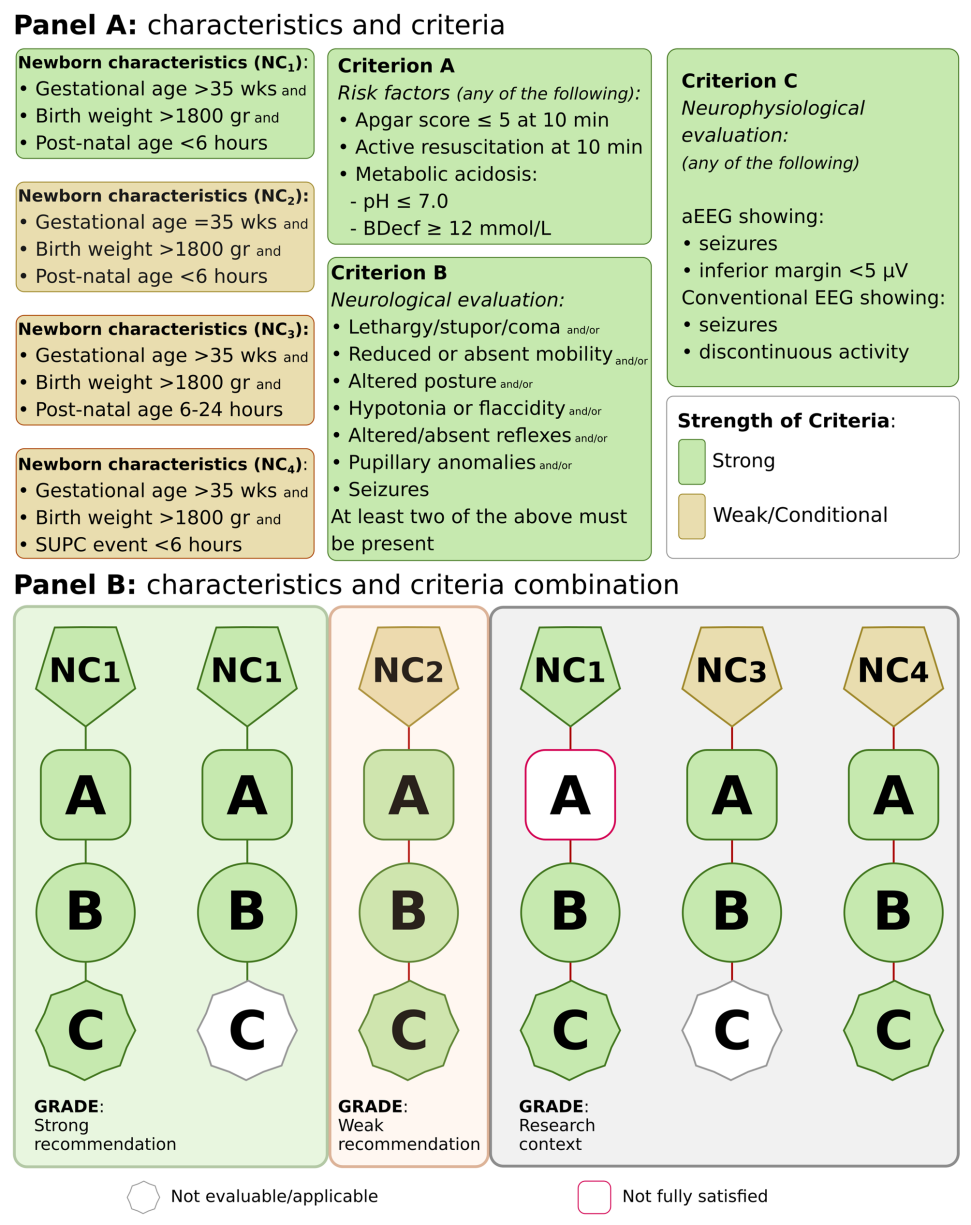
Criteria for therapeutic hypothermia recommendations. Panel a outlines newborn characteristics and criteria for eligibility, including risk factors (Criterion A), neurological evaluation (Criterion B), and neurophysiological assessment (Criterion C). Panel B depicts the combinations of these criteria leading to strong, weak, or research-context recommendations for initiating therapeutic hypothermia. The GRADE system (grading of recommendations, assessment, Development, and evaluation) is applied to determine the strength of each recommendation
- *Risk Factor Assessment*: for eligible newborns, the clinical significance of the asphyxia event (“*Criterion A*”) must be evaluated to identify those actually at risk of moderate to severe hypoxic-ischemic encephalopathy. If at risk, neurological and neurophysiological evaluations (“*Criteria B and C*”) determine whether therapeutic hypothermia is warranted. Unlike inclusion criteria, these risk factors show high consistency in the literature, all defined as “*Strong*”.
- *Therapeutic Hypothermia Initiation*: the strength of each above listed criteria is combined to formulate an overall “*Strong*” or “*Weak/Conditional*” recommendation for initiating therapeutic hypothermia or not. Additionally, conditions where there is insufficient evidence supporting the use of therapeutic hypothermia as standard therapy were identified and categorized as “*Research Context*”. These scenarios reflect situations in which current evidence is insufficient to support routine clinical use, and hypothermia should be evaluated exclusively within ethically approved research studies addressing clearly defined patient-important outcomes relevant to the present work.

The current clinical guidelines provide a step-by-step approach designed to assist physicians in quickly and effectively identifying newborns candidate to therapeutic hypothermia:

- *Inclusion Criteria (Newborn Characteristics)*: eligibility serves as the initial screening step. Newborns not meeting these criteria require no further assessment, even if a significant asphyxia event occurred. Notably, inclusion criteria in the available literature show significant heterogeneity. Thus, we classified different thresholds as “*Strong*” or “*Weak/Conditional*” criteria to proceed to further assessment, based on quality of available evidence, as summarized in Fig. 4.

**Fig. 5:**
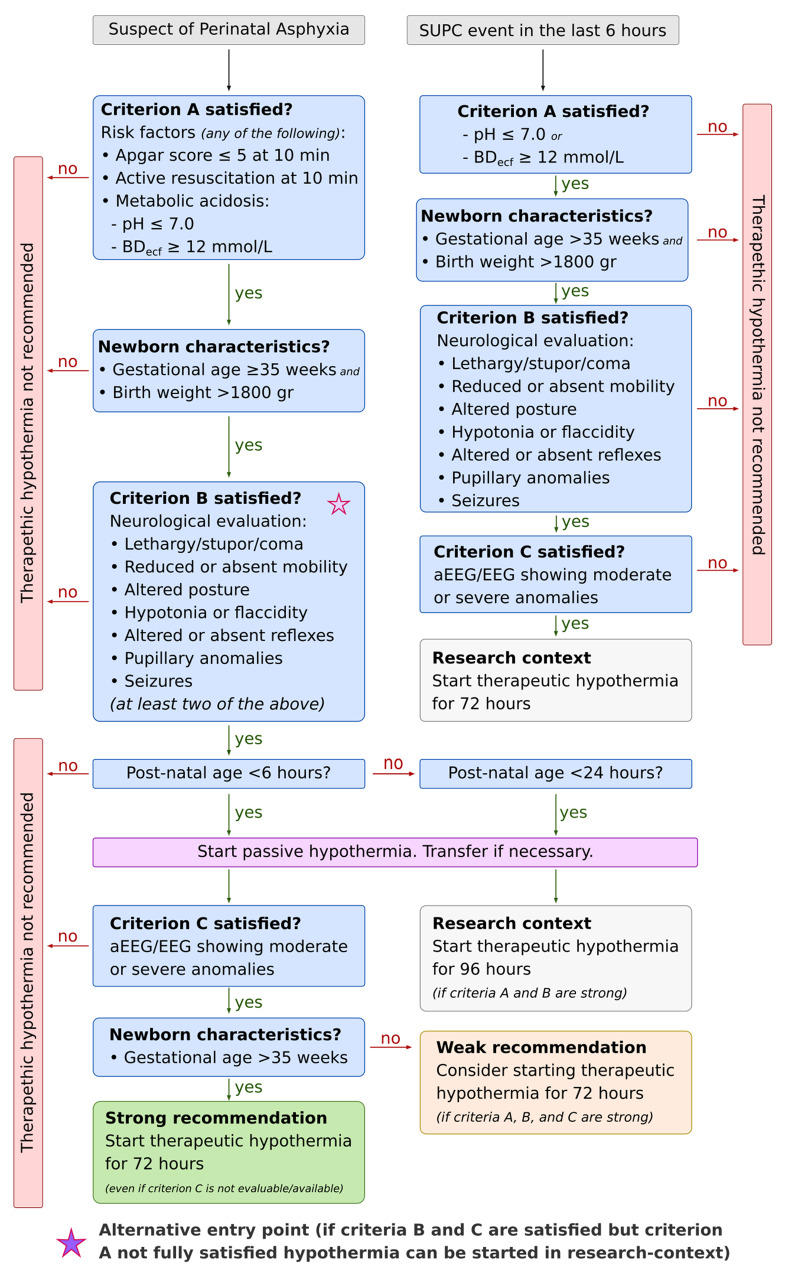
Flowchart for Neonatal therapeutic hypothermia eligibility. A decision algorithm guiding therapeutic hypothermia eligibility for neonates with suspected perinatal asphyxia or sudden unexpected postnatal collapse (SUPC). The stepwise evaluation includes: *Newborn Characteristics*: gestational age, birth weight, and time since asphyxia. *Criterion A*: presence of perinatal risk factors (e.g., apgar ≤ 5 at 10 min, metabolic acidosis, active resuscitation). *Criterion B*: neurological evaluation. *Criterion C*: neurophysiological findings (aEEG/EEG abnormalities). The strength of recommendation varies based on the combination of criteria met, ranging from strong recommendations to research-based settings

This structured approach, summarized in Figs. 4 and 5, ensures clear decision-making, aiding neonatologists in systematically assessing risk and clinical evidence when considering therapeutic hypothermia.

## Results

### Systematic review and clinical guidelines

A total of 8 RCTs including 1,843 newborns met the inclusion criteria after screening 344 non-duplicate records. Risk of bias assessment indicated that while most studies had low risk in randomization and intervention domains, concerns were identified regarding missing data and selective reporting, with some studies rated as high risk and consequently excluded. Overall, the studies were well-balanced, as the weighted RoB 2 assessment showed an equal distribution of medium and low risk studies, as reported in Fig. 3. Trials that nearly met the inclusion criteria but were excluded at the final screening stage are listed in the Supplementary Table “*Excluded Studies*”. For brevity and clarity, we provide here the key indications and recommendations. For a more detailed explanation of each section, please consult the Extended Results section in the Supplementary Materials. Details of selected studies are available in the Supplementary Table “*Included Studies*”.

#### Newborn characteristics

Eligibility criteria for therapeutic hypothermia are classified into two evidence strata - “*Strong*” and “*Weak/Conditional”* - according to the certainty of evidence supporting each criterion. Strong criteria apply to those with gestational age > 35 weeks, body weight > 1800 g, and postnatal age < 6 hours. H*igh-quality evidence* [10–13, 15]. Postnatal age > 6 hours [28] lowers the strength to “W*eak/Conditional”*, as illustrated in Fig. 4. Importantly, the certainty of evidence for newborns < 36 weeks gestation is extremely low [15, 29]. Accordingly, newborns at 35 weeks gestational age who meet criteria for peripartum asphyxia may undergo aEEG screening to stage encephalopathy, with a “*Weak/Conditional”* strength of recommendation. Similarly, the presence of a moderate/severe hypoxic-ischemic encephalopathy following sudden unexpected postnatal collapse is a “*Weak/Conditional*” criteria to proceed with the neurophysiological screening [5, 6, 30–33].

#### Criterion A: peripartum asphyxia

The criterion aims to promptly identify newborns at risk for peripartum asphyxia. Significant risk is indicated by severely impaired physiological adaptation at birth, defined as:

- Apgar score ≤ 5 at 10 minutes [10–13, 15]
- Need for active resuscitation (positive pressure ventilation with bag and mask, endotracheal tube, or other devices) at 10 minutes of life [10–13, 15]
- Metabolic acidosis, confirmed via arterial cord blood or neonatal blood gas analysis within 60 minutes, showing pH ≤ 7.0 [10–14, 18], or base deficit ≥ 12 mmol/L (in extracellular fluid) [15]

These factors, supported by *High-quality evidence*, indicate substantial risk.

#### Criterion B: neurological evaluation

Once a newborn is identified at risk for significant peripartum asphyxia, it becomes imperative to assess whether it has led to neurological impairment. A detailed neurological examination must be conducted in such cases, and considered altered if at least two of the following signs are present:

- Lethargy, stupor or coma
- Reduced or absent mobility
- Altered posture
- Hypotonia or flaccidity
- Weak, incomplete, or absent primitive reflexes
- Pupillary anomalies
- Seizures

The use of this criterion is supported by *High-quality evidence* [10, 12–15].

The neurological signs listed above in Criterion B reflect the clinical domains of the neurological examination included in the original Sarnat classification and their subsequent operationalization in later observational studies and randomized trials included in the present work [10–15, 28, 29, 34–36].

Neurological assessment is critical for eligible newborns and carries significant weight in determining overall therapeutic hypothermia eligibility. If neurological status is altered, aEEG/EEG evaluation (*Criterion C*) must proceed, even if *Criterion A* is unmet.

For newborns ≥ 36 weeks of gestational age requiring *Criterion C* evaluation, passive cooling must be initiated immediately by switching off warming devices and maintaining a rectal temperature of ~35 °C. *Moderate quality evidence. Strong recommendation* [7, 37–40].

If the birth center lacks therapeutic hypothermia capabilities, urgent transfer is mandatory [10, 12, 13]. Further details on stabilization and transportation are provided in the Chapter “*Stabilization and Emergency Transport*”.

#### Criterion C: neurophysiological evaluation

Neurophysiological monitoring via aEEG/EEG is the most accurate method for assessing neurological changes post-asphyxia in neonates [41–43]. Recording should last at least 30 minutes and, ideally, be performed before administering antiepileptics, analgesics, sedatives, or any drug affecting brain electrical activity [44–46].

- *Pathological aEEG*: defined by the presence of a lower margin < 5 µV, with an upper margin < 10 µV (severely abnormal trace) or ≥10 µV (moderately abnormal trace), and/or electrical seizures. *High-quality evidence. Strong recommendation* [10, 12, 13, 47].
- *Pathological EEG*: discontinuous background activity with inter-burst intervals < 10 seconds, no clear sleep-wake cycles (moderate anomalies); discontinuous background activity with inter-burst intervals 10-60 seconds, absent sleep-wake cycles (severe anomalies); marked attenuation of background amplitude with inter-burst intervals > 60 seconds (very severe anomalies); electrical seizures. *High-quality evidence. Strong recommendation* [13, 48–52].

Further details regarding the interpretation of conventional EEG and aEEG patterns are provided in the Supplementary Materials.

Whenever possible, aEEG monitoring should be continued until at least the end of the sixth hour of life in order to detect cases in which an initially normal tracing subsequently evolves into pathological patterns within the therapeutic window.

Importantly, aEEG traces may yield false negatives due to artifacts (e.g., muscle activity, high impedance). In such cases, conventional EEG should be obtained; if conventional EEG is unavailable due to organizational constraints, *criterion C* should be labeled as “*non-evaluable*”.

Further details on severity classification based on neurophysiological evaluation and aEEG artifacts identification are provided in Supplementary Materials.

#### Criteria combination

Therapeutic hypothermia is strongly recommended for newborns with strong baseline characteristics and strong *Criteria A and B* [10, 12, 13], even if *criterion C* is “*non-evaluable*” [11, 14, 15].

Therapeutic hypothermia may be beneficial, with a weak/conditional recommendation, in case of newborns of 35 weeks of gestational age, when Criteria A, B, and C are all strong. In such cases, therapeutic hypothermia may be considered after careful discussion with the parent or guardian of possible benefit and associated risk. Additionally, when Criterion A is not satisfied, or when baseline characteristics are classified as weak/conditional because the postnatal age exceeds 6 hours despite Criteria A and B being strong, or in cases of SUPC event when all the other criteria are strong, therapeutic hypothermia may be initiated only within a research context [15].

A graphical summary of criteria combinations and indications is provided in Fig. 4, with the operative algorithm outlined in Fig. 5.

#### Exclusion criteria

Some studies suggest potential exclusions, including terminal condition, major congenital anomalies, severe coagulation disorders, and intracranial hemorrhages [32, 53–55]. Available evidence confirms contraindications only for severe intracranial hemorrhages [32].

### Therapeutic hypothermia

#### Setting requirements

Therapeutic hypothermia requires a level II neonatal intensive care unit and a hub-spoke model for coordinated care between level I and II centers. A 1:1 or 1:2 nurse-to-patient ratio and 24/7 neonatologist in-house availability are essential [37, 56]. If an eligible newborn is born in a facility without therapeutic hypothermia, urgent transfer to a referral center is critical to meet treatment timelines.

#### Stabilization and Emergency transport

Transport for therapeutic hypothermia must prioritize cardio-respiratory and metabolic stability. While neonatal resuscitation [57, 58] and transport guidelines [59, 60] are beyond the scope of this work, we only focus on key points specific to these newborns:

- *Respiratory Support*: up to 50% of newborns with hypoxic-ischemic encephalopathy retain spontaneous respiration [56, 61–64]. Therefore, endotracheal intubation should be decided case by case, ensuring hyperoxia and hypocapnia are avoided, as both are associated with adverse outcomes [37, 62–70].
- *Temperature Control*: neurological outcomes depend on precise temperature regulation. Before initiating hypothermia, rectal temperature should be maintained at 35 °C, monitored continuously or every 15 minutes [37, 71–75]. If servo-controlled cooling is unavailable, passive hypothermia can be used. *High-level evidence. Strong recommendation* [7, 37, 39, 40, 73, 75–81].

#### Methods and devices

Therapeutic hypothermia can be implemented using two main strategies, both equivalent in efficacy [20]:

1. *Selective Head Cooling*: targets a rectal temperature of 34–35 °C, cooling only the head while maintaining mild systemic hypothermia [10, 14].
2. *Systemic Hypothermia*: cools the entire body to a rectal temperature of 33–34 °C [11–13, 15].

Continuous rectal temperature monitoring is required, with the probe placed 5–6 cm inside the anal orifice. Medication adjustments are crucial, as sedatives, analgesics, and muscle relaxants can lower body temperature, increasing the risk of over-cooling while hypothermia itself alters their pharmacokinetics, further elevating the risk of adverse events [56]. Devices play a key role in determining newborn outcomes, with emerging evidence highlighting the short- and long-term detrimental effects of various contaminants to which newborns are exposed, particularly during in-hospital procedures [82–84]. While identified RCTs have reported different devices for performing therapeutic hypothermia - especially in developing countries (e.g., gel packs, phase-change materials, cooling blankets, please refer to Supplementary Table) - ensuring neonatal safety remains paramount. Minimizing exposure to toxins and contaminants requires the use of authorized, rigorously controlled equipment. In this regard, servo-controlled systems are strongly recommended for both efficacy and safety [73, 75].

#### Newborn management

Effective pain and stress control is mandatory and significantly improves treatment outcomes. Non-pharmacological strategies include minimizing stimuli, optimizing positioning, promoting non-nutritive sucking, minimal enteral feeding, and both infant and family centered developmental care. *Moderate quality evidence. Strong recommendation* [85–87].

Sedative and anesthetic agents are known to have a detrimental impact on neonatal outcomes, when administered prior to birth, particularly in urgent situations that often precede the birth of asphyxiated newborns requiring therapeutic hypothermia [88, 89]. Achieving a proper balance between sedation, analgesia, and neuroprotection is both delicate and essential in this setting. Pharmacological management primarily involves opioid and benzodiazepines, requiring careful titration to prevent adverse effects. Emerging sedatives such as dexmedetomidine also play a significant role. *Moderate quality evidence. Strong recommendation* [12, 13, 85, 86, 90].

Seizure management in this patient population is critical, given the high incidence of seizures following perinatal asphyxia. Phenobarbital and phenytoin are first-line treatments, with additional options including benzodiazepines, lidocaine, topiramate, and levetiracetam. Due to literature heterogeneity, no precise recommendation can be provided [91–93].

Parental involvement is a critical aspect for improving neonatal outcomes. Perinatal asphyxia and therapeutic hypothermia are generally described as traumatic by parents, leading to separation anxiety and attachment difficulties [27, 94–97]. Clear and compassionate communication from the outset, provision of detailed explanations and written materials, regular updates, and active parental involvement in newborn care, are of central importance in this view. Encouraging parental touch and ensuring consistent nursing staff, alongside standardizing care protocols and offering personalized counseling, are also pivotal to alleviate parental stress. *Moderate level evidence. Strong recommendation* [97–99].

#### Side effects

Therapeutic hypothermia is safe when administered in well-equipped and trained intensive care units following established guidelines. However, three significant complications require close monitoring and prompt management: bradycardia, thrombocytopenia, persistent pulmonary hypertension, and adiponecrosis [18, 56, 87]. These are generally mild to moderate and resolve with minor interventions, ensuring the safety and efficacy of therapeutic hypothermia [18].

#### Rewarming

Rewarming begins after 72 hours of cooling, increasing temperature by 0.5 °C per hour over at least 4 hours. *High level evidence. Strong recommendation* [10–15].

During and after rewarming, continuous monitoring of rectal temperature, vital signs, arterial pressure, and aEEG/EEG is required for at least 4 hours, ideally extended to 12 hours. The rewarming phase carries a higher risk of seizures, particularly in cases previously controlled. If seizures occur, prolonging hypothermia for 4–12 hours may be necessary, depending on seizure characteristics and the clinical scenario. *Low level evidence. Weak/Conditional Recommendation* [60, 100, 101].

#### Neuroimaging

Magnetic resonance imaging (MRI) is the gold standard for assessing hypoxic-ischemic encephalopathy. Timely MRI scans are crucial for accurate diagnosis: early scans within a week post-event effectively detect the severity of damage using spectroscopy and diffusion-weighted imaging, while later scans provide a better assessment of damage extent through T1 and T2 sequences [102–105]. Early imaging differentiates neonatal encephalopathy types and helps inform medical decisions and parental counseling [106]. All newborns undergoing therapeutic hypothermia should receive brain MRI, preferably between 10 and 14 days of life to define lesion extent on T1/T2. When feasible, an early MRI may be performed at day 4–6 to maximize detection of diffusion restriction on DWI/ADC and to support earlier prognostication or when the clinical, neurophysiological, or ultrasound evaluation is suggestive of other causes of neonatal encephalopathy; a follow-up MRI at day 10–14 should be taken into consideration when there is a discrepancy between the early examination and the clinical condition of the newborn, or in the case of ambiguity of the first scan. In summary, earlier imaging (4–6 days of life) may be indicated in selected cases; however, repeating MRI at 10–14 days is recommended to allow a more accurate definition of the extent and pattern of brain injury.

#### Follow-up

Children treated with therapeutic hypothermia should be monitored until school age, similar to other high-risk populations. Neonatal hypoxic-ischemic encephalopathy is associated with reduced cognitive and psychomotor function, and behavioral problems. Developmental follow-up using evaluated tools for comprehensive neurocognitive and behavioral assessment are recommended at 18–24 months of age, with follow-up assessments until ages 6 to 7. *Moderate level evidence. Strong recommendation* [107–112].

Recent research suggests an integrated early assessment using MRI, general movements and neurological examination, within the first 5 months of life may aid early diagnosis and timely interventions. *Low level evidence. Weak/Conditional recommendation* [113].

### Future directions

Debate continues over expanding therapeutic hypothermia indications, particularly in:

- Mild cases
- Preterm infants
- Additional neuroprotective strategies

A detailed discussion is available in Extended Results section in the Supplementary Materials.

## Discussion

Through a systematic review of the available literature and GRADE assessment, we present operational guidelines for the application of therapeutic hypothermia in newborns with hypoxic-ischemic encephalopathy. These guidelines provide neonatologists with a structured, step-by-step approach to efficiently identify eligible newborns and ensure safe and effective implementation of therapeutic hypothermia in neonatal intensive care units.

Additionally, we outline setting requirements, stabilization and transport, and follow-up guidelines for therapeutic hypothermia. Each recommendation follows the GRADE methodology, with evidence quality and recommendation strength clearly presented to support clinical decision-making. Summarizing plots and algorithms further facilitate the immediate application of these guidelines in time-sensitive settings.

## Limitations and future directions

The present recommendations are based on a systematic review of the available literature conducted to provide a more comprehensive and updated discussion of the 2023 Italian Society of Neonatology guidelines. Nevertheless, some limitations should be acknowledged. First, although therapeutic hypothermia for moderate-to-severe hypoxic-ischemic encephalopathy is supported by multiple randomized controlled trials, many important clinical questions remain insufficiently addressed by high-quality evidence. Several key aspects of clinical management - including the optimal criteria for early neurological evaluation, the presence or absence of a sentinel event, the duration of aEEG monitoring, and the management of specific subgroups such as infants born at 35 weeks gestational age - are supported mainly by observational studies, secondary analyses of clinical trials, or expert consensus rather than randomized trials dedicated to these specific aspects. In addition, the available studies show heterogeneity in inclusion criteria, neurological assessment methods, and electrophysiological definitions, which may influence the comparability of results across trials. Finally, many of the pivotal trials were conducted more than a decade ago and in specific healthcare settings, potentially limiting the generalizability of their findings to contemporary neonatal practice. For these reasons, ongoing research and updated evidence synthesis will be essential to refine these recommendations and inform future revisions of the guidelines.

Important issues will need to be addressed in the near future, starting with the criteria used for neurological evaluation. In the main supporting trials, altered consciousness (lethargy, stupor, or coma) was frequently listed among the mandatory inclusion criteria used to define moderate-to-severe hypoxic-ischemic encephalopathy [10, 13, 14, 34]. However, the enrolled cohorts still included infants with mild forms of hypoxic-ischemic encephalopathy, which is not compatible with lethargy, stupor, or coma according to the Sarnat definition [114, 115]. This discrepancy suggests operational variability in early neurological assessments, likely reflecting both the evolving nature of clinical signs and the inter-observer variability inherent to a purely observation-based clinical evaluation. Moreover, the Sarnat definition of “*lethargy*” is inherently subtle and therefore difficult to standardize in clinical practice. In the original description, lethargy was defined as “*a state of slightly delayed but complete response to stimuli, and slightly increased threshold for eliciting such responses, and decreased amount of spontaneous movement*” [34]. Such a nuanced definition makes consistent operationalization challenging. Accordingly, the level-of-consciousness item has demonstrated the lowest sensitivity (43%) in early neurological evaluations [116]. To minimize false negatives within the therapeutic window, we therefore operationalized Criterion B as the presence of at least two neurological signs, which prompts aEEG screening rather than immediate initiation of hypothermia. Within this framework, occasional false positives only result in monitoring and reassessment, whereas in case of false negatives this approach could provide timely treatment. This sensitivity-oriented approach helps preserve eligibility within the integrated diagnostic algorithm while avoiding reliance on a rigid single-item requirement for altered consciousness. From a research perspective, we strongly advocate for dedicated evidence-synthesis studies aimed at systematically categorizing the different approaches to neurological assessment used across trials and evaluating whether these methodological differences influence the reported effectiveness of therapeutic hypothermia. Potential revisions to this important Criterion B will be considered in the next update of the guidelines, with specific efforts directed toward clarifying this critical issue.

Infants born at 35 weeks gestational age who develop significant hypoxic-ischemic encephalopathy represent another important issue. In this population, interpreting the neurological examination during the first hours of life remains difficult, as it is often challenging to distinguish findings related to prematurity from those attributable to hypoxic-ischemic encephalopathy. A recent randomized trial found no evidence of either benefit or harm of therapeutic hypothermia in this population, although the authors noted that formal analyses to determine the probability of benefit or harm were not performed because of the small sample size [117]. In the present guidelines we suggest that these newborns may undergo aEEG screening to stage encephalopathy when peripartum criteria for asphyxia are met; however, initiation of therapeutic hypothermia should be approached with extreme caution. In such cases, treatment should be limited to level III NICUs with continuous monitoring and experienced teams, following a multidisciplinary evaluation of the individual clinical scenario (including the presence of an acute sentinel event, precise gestational age and its certainty, birth weight, aEEG findings, and exclusion of intracranial hemorrhage), and shared decision-making with informed parental consent, acknowledging the very low certainty of available evidence for this gestational age. Further studies specifically addressing this population are urgently needed, and potential revisions of this recommendation will be considered in the next update of these guidelines.

Another important issue that will be considered in the next update of the guidelines concerns the optimal duration of aEEG monitoring. In the present recommendations, we indicate that at least 30 minutes of recording should be obtained, while suggesting that monitoring should be continued until up to six hours of life whenever feasible. In future revisions of the guidelines, we will evaluate whether extending aEEG monitoring to the entire six-hour therapeutic window should become a standard requirement, in order to identify cases in which an initially normal tracing subsequently evolves into a pathological pattern. Further research specifically addressing the optimal duration of early aEEG monitoring is urgently needed to better inform future recommendations.

Finally, the conditions in which therapeutic hypothermia may be initiated within a research context (including cases in which Criterion A is not fully satisfied, postnatal age between 6 and 24 hours, or following a SUPC event) will represent another important aspect to be addressed in the next update of the current guidelines. Evidence supporting the use of therapeutic hypothermia in these situations remains extremely limited and, more importantly, is difficult to generate, as randomized trials are unlikely to be feasible due to ethical considerations and the fortunately extremely low number of cases. In this context, meaningful evidence may emerge from observational studies, case series, and coordinated data collection initiatives. In particular, the development of national or international registries may represent a valuable strategy to systematically collect and analyze these rare cases at a larger scale. In this regard, the Italian Society of Neonatology is currently developing such a registry, which we hope will contribute to strengthening the evidence base for the next revision of these guidelines.

## Conclusion

These guidelines provide a structured protocol for implementing therapeutic hypothermia, based on a systematic literature review and adhering to the GRADE methodology. Designed for seamless clinical integration, this document includes summarizing plots and algorithms to enhance usability in real-world practice. Periodic updates will ensure alignment with the latest scientific advancements.

## Electronic supplementary material

Below is the link to the electronic supplementary material.

Supplementary material 1

Supplementary material 2

## Data Availability

All data relevant to the study are publicly available and included within the article or provided as supplementary information.
