# Supplementary material for: Therapeutic hypothermia in newborns: evidence-based guidelines from a systematic review
Source: Ital J Pediatr. 2026 Apr 27;52:103. doi: 10.1186/s13052-026-02266-x (PMC13255436; doi:10.1186/s13052-026-02266-x)
Supplement: Supplementary file 1 — Supplementary material 1 [file 13052_2026_2266_MOESM1_ESM.docx]

**Extended Methods**

**Research Strategy**

A systematic literature search was conducted across multiple databases, including MEDLINE (via OVID), Scopus, Cochrane Library, Embase, and Web of Science (WOS), to identify relevant randomized controlled trials (RCTs) on neonatal hypothermia focusing on or reporting neurodevelopmental outcomes. The search strategy was developed in consultation with a medical librarian and tailored for each database using Medical Subject Headings (MeSH) terms, keywords, and Boolean operators. The search terms targeted three main themes:

- **Population**: Newborns (e.g., "*Infant, Newborn*", "*Premature Infant*", "*Small for Gestational Age*", "*Low Birth Weight*" and related terms).
- **Intervention**: Hypothermia-related therapies (e.g., "*Hypothermia*", "*Therapeutic Hypothermia*", "*Cooling*", and "*Freezing*").
- **Outcomes**: Neurodevelopmental disorders and cognitive development (e.g., "*Neurodevelopmental Disorders*", "*Cognitive Impairment*", "*Psychomotor Development*", "*Autism Spectrum Disorder*", and "*Learning Disabilities*").
- **Study Design**: Clinical trials, with a focus on randomized controlled trials (e.g., "*Randomized Controlled Trial*", "*RCT*", "*Clinical Trial*").

The search strings were customized for each database as follows:

- *MEDLINE (OVID)*: A combination of MeSH terms and free-text keywords, with filters applied for RCTs where applicable.
- *Scopus & Embase*: Searches were conducted using controlled vocabulary and keyword variations in the abstract, title, and keyword fields.
- *Cochrane Library*: Searches were performed using MeSH descriptors with an "explode" function to include all subcategories.
- *Web of Science (WOS)*: The topic search function was used to retrieve results based on key terms.

No language or date restrictions were applied. The final search was conducted on January 5, 2024, and results were exported for manual screening. We included only RCTs that explicitly reported inclusion criteria and assessed neurodevelopmental outcomes in newborns undergoing therapeutic hypothermia for hypoxic-ischemic encephalopathy at birth or sudden unexpected postnatal collapse (SUPC) following birth. Studies were considered even if neurodevelopmental outcomes were not the primary outcome under investigation, provided they were reported in the study results. The primary outcome was neurodevelopment at 18-24 months, assessed with validated tools or equivalent composite measures of cognitive and motor function. RCTs without an assessment in this window were excluded from the primary evidence base and GRADE certainty. Only trials scoring low risk or some concerns on the RoB2 scale were considered, excluding high risk RCTs. We evaluated therapeutic hypothermia as a standalone neuroprotective therapy (TH vs standard care). Trials testing adjunctive pharmacologic agents in addition to TH (e.g., erythropoietin, melatonin, xenon, topiramate, allopurinol) were excluded from the primary evidence base because they address combination strategies rather than the effectiveness/indications of TH itself. Such trials are listed in the Supplementary Table “*Excluded Studies*” and briefly summarized for context. Detailed search strategy and results for each research question are reported in Supplementary Figure 1.

***GRADE Quality of Evidence***

According to the GRADE methodology (1), the quality of evidence is classified based on the type of study and a methodological assessment. The classifications are as follows:

- *High*: evidence from randomized controlled trials or systematic reviews of randomized trials. Further research is very unlikely to change our confidence in the estimated effect.
- *Moderate*: evidence may include lower quality randomized trials, systematic reviews, or high-quality observational studies. Further research may have a significant impact on our confidence in the estimated effect and may change the estimate.
- *Low*: evidence from case-control or cohort studies. Further research is likely to have an important impact on our confidence in the estimate of effect and is likely to change the estimate.
- *Very Low*: evidence includes observational studies with high risk of bias. The estimate of effect is very uncertain, and further research is very likely to change the estimate.

This classification helps guide clinical decision-making by indicating the level of confidence in the available evidence and the likelihood that further research could alter these findings.

***GRADE Strength of Recommendation***

The strength of a recommendation in the GRADE system combines the quality of evidence, risk/benefit analysis, patient values, and resource considerations, assessing whether the benefits outweigh the potential adverse effects. The key benefits of therapeutic hypothermia include reduced mortality, psychomotor delay, neurodevelopmental impairment, and neonatal seizures, while adverse effects mainly include significant clinical complications. GRADE categorizes recommendations into:

- *Strong Recommendation*: when, based on high or moderate quality evidence, the desirable effects of following the recommendation clearly outweigh the undesirable effects. This typically becomes the standard of care.
- *Weak or Conditional Recommendation*: when the benefits likely outweigh the risks, but the evidence is low or very low, making the balance less certain. This requires careful consideration and often involves discussions with the healthcare team and the newborn caregivers to tailor the approach to individual patient needs and contexts.

In essence, a “*strong”* recommendation suggests broad application, while a “*weak/conditional”* recommendation advises a more individualized approach.

In situations where pathophysiological and clinical evidence suggests potential benefits of hypothermia but lacks strong evidence to definitively support its efficacy - a condition known as "equipoise" - hypothermic treatment should be considered primarily within a clinical “*research context*”. According to GRADE, “Only-in-research recommendations will be appropriate when three conditions are met: (1) there is insufficient evidence supporting an intervention for a panel to recommend its use; (2) further research has a large potential for reducing uncertainty about the effects of the intervention; and (3) further research is deemed good value for the anticipated costs. The research recommendations should be detailed regarding the specific research questions that investigators should address, particularly which patient-important outcomes they should measure. The recommendation for research may be accompanied by an explicit strong recommendation not to use the experimental intervention outside of the research context.” Accordingly, these scenarios are explicitly classified as “Research Context,” indicating that therapeutic hypothermia should be evaluated exclusively within ethically approved research studies addressing clearly defined patient-important outcomes relevant to the present work. In line with GRADE guidance, this designation is accompanied by a strong recommendation against the use of therapeutic hypothermia outside of such research settings. These scenarios reflect situations in which current evidence is insufficient to support routine clinical use, but where further research has the potential to meaningfully reduce uncertainty and is considered a valuable use of resources. This approach requires that the decision to proceed with hypothermia is made with full transparency and strict adherence to ethical standards. Comprehensive informed consent must be obtained, clearly emphasizing the experimental nature of the treatment under these circumstances.

***Implementing Therapeutic Hypothermia: Safety and Cost Considerations***

Implementing the recommendations for therapeutic hypothermia is safe, marked by a beneficial safety profile and a low incidence of complications. This treatment necessitates appropriate setting, equipment, monitoring, and training as specified in the "Setting Requirements" section. Adoption of these guidelines should not incur additional costs beyond those already associated with the medical devices and drugs used in neonatal intensive care units.

**Extended Results**

***Search Strategy***

The literature search identified a total of 402 unique records across all databases. The breakdown of search results per database is as follows:

- MEDLINE (OVID): 312,234 results for RCT-related terms, 374,132 for neurodevelopmental disorders, 130,936 for hypothermia, and 2,284,196 for neonatal-related terms. The final combined search yielded 73 studies meeting inclusion criteria.
- Scopus: The intersection of neonatal, hypothermia, neurodevelopmental, and RCT-related searches resulted in 206 studies.
- Cochrane Library: The combination of search terms retrieved 55 relevant studies.
- Embase: A total of 33 studies were identified after applying the combined search filters.
- Web of Science (WOS): A total of 35 studies were retrieved.
- Following deduplication and screening, 8 studies met the eligibility criteria and were included in the systematic review.

Figure 2 reports the PRISMA flowchart with records for each selection step, and Supplementary Figure 1 reports the exact search strategy and results identified for each database.

***3.1.1. Newborn characteristics***

Newborn characteristics categorize potential candidates for therapeutic hypothermia into “*Strong*” or “*Conditional/Weak*” criteria to proceed with further assessment. A strong indication is assigned to all newborns with a gestational age of over 35 weeks, a body weight exceeding 1800 grams, and a post-natal age of less than 6 hours; these candidates are eligible for therapeutic hypothermia, supported by *high-quality evidence* (2–6).

The absence of each indicator modifies the strength of the criteria to weak:

- A gestational age of exactly 35 weeks (from 35+0 to 35+6 post-menstrual age), with weight and post-natal age criteria met, can be considered for hypothermia only in *research setting* (6).
- A post-natal age between 6 to 24 hours after birth, with respected gestational age and weight, leads to a *very low quality of evidence and a weak/conditional recommendation (7)*.

Similarly, a SUPC event occurring within the last 6 hours in a newborn with more than 35 weeks of postmenstrual age and a birth weight over 1800 grams also results in a *weak/conditional recommendation* to proceed with further assessment (8–13).

These newborn characteristics are summarized in Figure 4.

***3.1.2. Criterion A: Peripartum Asphyxia***

The rationale behind defining this criterion is to establish a clear and rapid assessment framework for identifying newborns at risk of peripartum asphyxia. This assessment is crucial when any of the following criteria are met:

- An Apgar score of 5 or less at 10 minutes of life. *High-quality evidence. Strong recommendation*. (2–6)
- Active resuscitation at 10 minutes of life, involving interventions such as an endotracheal tube, facial mask, or other non-invasive procedures for cardiorespiratory support. *High-quality evidence. Strong recommendation*. (2–6)
- Fetal or neonatal metabolic acidosis, evidenced by a blood gas analysis from arterial cord blood or from the newborn themselves within 60 minutes of life, showing either:
  - pH of 7.0 or lower *High-quality evidence. Strong recommendation*. (2–5,14,15)
  - extracellular fluid base deficit of 12 mmol or greater. *High-quality evidence. Strong recommendation*. (6)

Further information on blood gas analysis execution and interpretation are provided in Blood Gas Analysis section. Criterion A is graphically summarized in Figure 4, panel A.

***3.1.3. Criterion B: Neurological Evaluation***

After identifying newborns at risk for peripartum asphyxia, it becomes imperative to assess whether it has led to significant and observable neurological impairment. This evaluation is conducted through a detailed neurological examination of the newborn, which should be considered abnormal if at least two of the following are detected:

- Lethargy, stupor or coma
- Reduced or absent mobility
- Altered posture
- Hypotonia or flaccidity
- Weak, incomplete, or absent primitive reflexes
- Pupillary anomalies
- Seizures

This approach results in a *High-quality evidence. Strong recommendation* (2,4–6,14).

The neurological evaluation is a crucial step for eligible newborns and it has a very strong weight on the overall indication. When there is evidence of a significantly altered neurological status, this evaluation must be prioritized. If criterion A is unmet, re-evaluation must consider possible alternative scenarios, such as:

- Errors in the collection of cord blood gas analysis (please refer to “*Blood Gas Analysis*” section below). In cases of doubt, perform a blood gas analysis on the newborn themselves within 60 minutes of life.
- A prolonged or partial form of asphyxia, where metabolic compensation may have already occurred at birth. (16)

In such cases, it is mandatory to proceed further with EEG/aEEG evaluation (criterion C), as graphically summarized in Figure 4.

In all newborns for whom criterion C evaluation is indicated, the following steps should be promptly initiated:

- *Passive Cooling*: once newborn is cardio-respiratory stable, avoid active warming of the newborn by turning off warming devices and maintaining a rectal temperature of approximately 35°C. *Moderate quality evidence. Strong recommendation*. (17–21)
- *Newborn Transfer*: if the birth center is not equipped to perform therapeutic hypothermia, transfer the newborn to a Level II referral center where the necessary care can be provided (2,4,5). Further details on stabilization and transportation procedures for neonates eligible for therapeutic hypothermia are provided in the relevant chapter “*Stabilization and Emergency Transport*”.

It is crucial that the rectal temperature of the newborn is accurately measured at 5-6 cm from the anal orifice using appropriate thermometers. Rectal temperature monitoring should be continuous or, at a minimum, conducted every 15 minutes whenever the chance of asphyxia exists (18,22–25). It is important to note that axillary temperature measurements cannot substitute for rectal temperature monitoring in this setting (22,23,26).

***3.1.4. Criterion C: Neurophysiological Evaluation***

Neurophysiological monitoring is the most precise method for assessing neurological alterations following asphyxia and plays a central role in neonatal patients, where neurological signs may be actively evolving (27–29). The aEEG/EEG should be recorded for at least 30 minutes and preferably before the administration of any antiepileptic, analgesic, or sedative therapy, as these drugs can reduce the voltage of the recorded electrical activity (30–32). In case aEEG is used, the evaluation should always be paired with the visualization of the underlying raw EEG trace.

The aEEG trace is deemed pathological if electrical seizures are present or if the lower margin falls below 5 microvolts. In the latter scenario, traces can be classified as highly pathological if the upper margin is below 10 microvolts and moderately pathological if it is above 10 microvolts. *High-quality evidence. Strong recommendation*. (2,4,5,33)

The raw EEG trace is considered pathological if it shows electrical seizures. Severity can be further differentiated based on the duration of inter-burst intervals and additional criteria:

- *Moderate anomalies*: discontinuous background activity with inter-burst intervals of less than 10 seconds, no clear sleep-wake cycles.
- *Severe anomalies*: discontinuous background activity with inter-burst intervals ranging from 10 to 60 seconds, absent sleep-wake cycles, and a significant reduction in trace amplitude (below 10 microvolts).
- *Highly severe anomalies*: an inactive or highly discontinuous trace with inter-burst intervals exceeding 60 seconds.

All such cases are to be considered pathological. *High-quality evidence. Strong recommendation*. (5,34–38) as graphically summarized in Figure 4, panel A.

It is important to note that the aEEG trace can yield false positives if artifacts are present. In such instances, criterion C should be labeled as “*non-evaluable*”. When classifying the aEEG trace, it is essential to exclude artifacts, which may lead to an erroneous overestimation of normal aEEG voltage, thereby reducing its sensitivity in detecting hypoxic-ischemic brain injury. These artifacts often originate from muscle activity rather than neuronal activity. Such artifacts have been reported in up to 15% of term neonates with clinical signs of moderate-to-severe hypoxic-ischemic encephalopathy when recordings were performed within the first six hours of life, particularly in neonates undergoing passive hypothermia (39–41). This artifact figure reported in the literature reflects experience with single-channel aEEG in the first six hours, particularly during passive hypothermia; generalizability to multi-channel systems is limited. To address this issue, it is necessary to analyze the conventional EEG trace alongside the aEEG band, assessing wave frequency and morphology. Sharp, monomorphic waves with a frequency often exceeding 10 Hz should raise suspicion of artifacts. The use of a fifth reference electrode may help reduce the impact of these muscle artifacts. Additional strategies to mitigate artifacts include:

- Gradually increasing the neonate temperature if it is below 35°C to reduce muscle artifacts.
- Complementing neurophysiological evaluation with raw EEG or video EEG.
- Referring the neonate to a level II center for aEEG evaluation.
- Extending the aEEG recording. The absence of cyclicity during this period may suggest a pathological trace, although this criterion has low specificity since cyclicity typically appears around seven hours of life.

There is no evidence supporting the use of melatonin as a sedative to reduce muscle artifacts during aEEG/EEG recordings. The American Academy of Neurophysiology recommends its use for pediatric EEG evaluation but not for neonatal EEG (42) . No sedative effect were identified with low-doses of oral melatonin (0.5 mg/kg) in neonates with hypoxic-ischemic encephalopathy (43). No data are available on its sedative effects at higher doses (44).

***3.1.5. Criteria Combination***

Based on the potential combinations of single strong/weak criteria outlined earlier, it can be summarized that therapeutic hypothermia is strongly recommended for all cases that exhibit strong baseline newborn characteristics (gestational age >35 weeks, birth weight >1800 grams, and post-natal age <6 hours) and strong criteria A and B, with criterion C being either strong (2,4,5) or non-applicable (3,6,14).

Conversely, a research-context recommendation is given for:

- Cases with strong baseline characteristics and strong criteria B and C, but the absence of criterion A.
- Cases with weak/conditional baseline characteristics due to a post-natal age of more than 6 hours, coupled with strong criteria A and B, even if criterion C is non-evaluable (7).
- SUPC events that occurred less than 6 hours ago in newborns with a gestational age of more than 35 weeks, a birth weight of over 1800 grams, and strong criteria A (taking into account blood gas tests conducted within an hour of the event or the need for cardio-respiratory support), B, and C. (8–13)

Cases with strong criteria A, B and C, but with exactly 35 weeks of gestational age (35+0 to 35+6) can be considered for therapeutic hypothermia under Weak/Conditional recommendation.

All possible combinations and the overall strength of recommendations are graphically summarized in Figure 4, panel B, while the summary operative algorithm is provided in Figure 5.

***Blood Gas Analysis***

The evaluation of blood gas values is crucial for diagnosing intrapartum asphyxia, a condition characterized by impaired gas exchange that leads to progressive hypoxia, hypercapnia, and significant metabolic acidosis. The presence of acidosis in fetal intrapartum, cord, or early neonatal blood gas analysis is one of the four essential criteria for associating an acute intrapartum event with cerebral palsy (45). The American College of Obstetricians and Gynecologists and the American Academy of Pediatrics recommend umbilical arterial and venous blood gas analysis in cases of an Apgar score below 7 at 5 minutes, preterm birth, intrauterine growth restriction, abnormal fetal heart rate tracing, maternal thyroid disease, intrapartum fever, or multiple gestations (46). Similarly, the National Institute for Health and Clinical Excellence advises performing this analysis when the Apgar score at 1 minute is ≤5 (47).

To ensure accurate sample origin, paired cord blood gas analysis should be performed, with samples collected from both the umbilical artery and vein. Sampling errors have been reported up to ~40% of cases (48–50), often due to both samples being drawn from the umbilical vein, resulting in similar values. Confirmation that the sample is arterial requires distinct blood gas values, where the umbilical arterial sample shows a lower pH and higher pCO₂ compared to the venous sample. When the reliability of cord blood samples is uncertain, neonatal blood gas analysis should be performed as soon as possible, ideally within the first 60 minutes after birth.

Blood collection should follow a double clamping technique, initially on the fetal side and then on the placental side, preferably before placental delivery. If delayed cord clamping has occurred, immediate sampling is necessary. In cases where cord blood collection is not feasible due to technical or logistical reasons, a neonatal blood sample (arterialized capillary, arterial, or venous) must be obtained within the first hour of life. If multiple postnatal samples are available, the one collected closest to birth should be used for *criterion A* assessment (51).

Some randomized controlled trials have included neonates with a pH <7.10 in therapeutic hypothermia protocols (52). An observational study (53) found that approximately 10% (34/327) of neonates with a pH between 7.00 and 7.10 develop moderate-to-severe hypoxic-ischemic encephalopathy. Additional studies have identified 7.1 as a critical pH threshold, below which the risk of encephalopathy and/or mortality increases significantly (53–55). The base deficit in extracellular fluid should also be considered, as it provides a more accurate representation of the metabolic component of acidosis. While umbilical arterial CO₂ correlates positively with blood base deficit, it does not show the same correlation with the extracellular fluid deficit, which is a more reliable indicator of metabolic acidosis (56).

***Stabilization and Emergency Transport***

Newborns eligible for therapeutic hypothermia who are born at centers where such treatment is unavailable must be promptly identified and transported to facilities where the treatment can be administered. The primary goal of transferring newborns is to maintain cardio-respiratory and metabolic stability both before and during transport. In this context, effective communication between the birth center and the referral center is essential to determine whether to delay or immediately perform certain maneuvers, depending on the clinical condition of the newborn. While neonatal resuscitation (57,58) and transport (59,60) guidelines are publicly available and beyond the scope of this work, we will focus on highlighting key points regarding respiratory and temperature assistance specific to these newborns.

Respiratory assistance is crucial for newborns with hypoxic ischemic encephalopathy, with 30 to 50% of such newborns maintaining an autonomous respiratory drive (61–65). Therefore, the decision to stabilize airways through endotracheal intubation should be made on a case-by-case basis by the attending physician. Importantly, to prevent hyperoxia and worsening neurological damage due to oxidative stress, it is essential to accurately and continuously regulate the inspired fraction of oxygen based on transcutaneous saturation levels, during both acute resuscitation and subsequent stabilization and transport (18,66–70). Furthermore, perinatal asphyxia is strongly associated with metabolic acidosis, leading to a tendency towards hyperventilation in affected newborns. This, combined with reduced metabolism and carbon dioxide production, often results in a strong tendency toward hypocapnia, which is linked to poor neurological outcomes in newborns with hypoxic ischemic encephalopathy that should be avoided (63–65,71). The mechanisms involved include cerebral vasoconstriction, reduced haemoglobin oxygen delivery to tissues (72), neuronal hyper-excitability due to GABA suppression (73), and nuclear DNA fragmentation (74). Additionally, hyperoxia with an arterial oxygen pressure above 200 mmHg further exacerbates poor neurological outcomes (75).

Precise temperature regulation is crucial for patients undergoing therapeutic hypothermia and is a key determinant of neurological outcomes. During transport, the newborn should be maintained at a rectal temperature of 35 degrees Celsius, monitored at least every fifteen minutes if continuous monitoring is unfeasible (18,22,24,25,76,77). Temperature maintenance can be achieved through the following methods:

- *Portable Servo-Controlled Systems*: these systems offer significant advantages by reaching the target temperature more quickly and maintaining it consistently, greatly reducing the risk of over-cooling. Furthermore, initiating therapeutic hypothermia during transportation has been shown to be associated with better outcomes (25,77–81). These systems should be preferred over passive methods. *High-level evidence. Strong reccomendation*.
- *Passive Hypothermia*: this method involves turning off warming sources (23) (*Low level evidence. Conditional recommendation*). The use of ice-filled packs should be avoided due to their risk of severe over-cooling, which can occur in up to 35% of cases (82). Warming devices should be readily available for use in case of over-cooling, but the primary focus should be on avoiding hyperthermia. *High-level evidence. Strong reccomendation*. (17,18,20,21)

***Newborn Management***

Newborns undergoing therapeutic hypothermia require close monitoring and support in a well-equipped intensive care unit, akin to other clinically unstable and life-threatening conditions. Several aspects need heightened vigilance and specific care, given the specific characteristics of therapeutic hypothermia and affected newborns fragile status. Here is a brief presentation of the most critical care elements:

- *pain and stress control*: therapeutic hypothermia is a stressful treatment which requires proper monitor and control of pain and stress. Effective monitoring and management of pain and stress are essential. Non-pharmacological interventions play a central role and have demonstrated efficacy without adverse effects in this context (83,84). *Moderate quality evidence. Strong recommendation*. Key strategies include minimizing external stimuli (such as light and noise), limiting handling, frequent postural changes to reduce the risk of adiponecrosis (85), promoting non-nutritional sucking and minimal enteral feeding, and fostering family-centered care. The use of sedative and anesthetic agents has been linked to adverse neonatal outcomes, as newborns are highly susceptible to the effects of medications that are typically well-tolerated in adults. Their immature metabolic and excretory systems, coupled with the unique physiology of the neonatal brain, significantly increase the risk of drug accumulation and neurotoxic effects. Notably, evidence has demonstrated that poor neonatal outcomes can occur even when these agents are administered prior to birth. This concern is particularly pronounced in urgent obstetric situations, such as emergency cesarean sections, which frequently precede the delivery of asphyxiated newborns requiring therapeutic hypothermia (86,87). Given these risks, as well as the delicate clinical status of newborns undergoing therapeutic hypothermia, a meticulous approach to sedation, analgesia, and neuroprotection is essential to optimizing neonatal outcomes. Striking the right balance between these factors is particularly challenging in this setting, necessitating careful selection and titration of agents to minimize fetal and neonatal exposure to potentially harmful drugs while ensuring both maternal and neonatal stability. In this view, while definitive guidelines are lacking in the literature, opioids and benzodiazepines are commonly reported for use during therapeutic hypothermia (88,89). Continuous infusion of opioids is the most widely adopted strategy. *Moderate quality evidence. Strong recommendation*. (4,5,83,84) However, no clear differences in outcomes were observed (90). Fentanyl, for example, can be initiated with a bolus of 1-2 mcg/kg followed by a maintenance infusion of 0.5-1 mcg/kg/h, adjusted based on the newborn response. Special attention must be paid to the potential accumulation of opioids due to decreased metabolism during hypothermia, which can increase the risk of adverse events. Infusion rates should not exceed 10 mcg/kg/h of morphine equivalent, as higher dosages have been shown to reach toxic levels during therapeutic hypothermia (91,92). Dexmedetomidine, an alpha-2 agonist, is increasingly considered for its safety profile and potential advantages, such as stable metabolism during hypothermia, neuroprotective properties, shorter weaning times from mechanical ventilation, and quicker progression to full enteral feeding (93–100). It can be used at infusion rates of 0.2-0.5 mcg/kg/h, with careful monitoring for major side effects like bradycardia and hypotension. Dexmedetomidine may be used either as monotherapy or in combination with opioids. *Low quality evidence. Conditional recommendation*.

- *seizure control*: seizures are a common sequel of perinatal asphyxia, yet there is no consensus on the optimal treatment approach. Most protocols and the available literature suggest the use of phenobarbital and/or phenytoin as first-line agents. These may be supplemented with benzodiazepines, lidocaine, topiramate, and levetiracetam. This approach is based on their efficacy in managing neonatal seizures, though treatment decisions often depend on the specific clinical scenario and the individual response of each newborn to these medications, making impossible to provide unique recommendation. (101–103).

***Family Care***

Recent evidence increasingly demonstrates that the development of newborns in critical care is significantly shaped by their relationships with their families and by the resilience of these families themselves (104–106), whose support is crucial during therapeutic hypothermia (107). Perinatal asphyxia and subsequent therapeutic hypothermia are often reported by parents as traumatic experiences, particularly due to the pale, shivering, and stiff appearance of the newborn during treatment, which can elicit separation anxieties and negatively impact attachment (108). To mitigate parental stress and enhance neonatal outcomes, the following recommendations can be made. *Moderate level evidence. Strong recommendation*. (108–110)

- *Informing Parents*: Parents should be extensively and effectively informed about the clinical situation and the role of therapeutic hypothermia in neonatal asphyxia from the outset, in order to aid their understanding of the treatment, its rationale, and necessity.
- *Detailed Explanation of Treatment*: Clearly explain each treatment and rewarming step, and the subsequent observational stay. Providing written materials can be helpful.
- *Effective Communication*: Ensure effective and prompt communication between obstetrical and NICU personnel to facilitate coordinated care.
- *Continuous Updates*: Keep parents constantly and effectively updated using direct, honest, and compassionate communication.
- *Simplifying Communication*: Avoid technical terms and complex phrasing when communicating with parents to ensure clarity and understanding.
- *Parental Care Involvement*: Involve parents in the care of the newborn, such as changing diapers, and allow them to stay in the same room with their baby to foster bonding and reduce stress.
- *Nurse Continuity*: Prioritize continuity in nursing staff in the NICU to help families become familiar with caregivers, enhancing trust and communication.
- *Standardize Care Protocols*: Standardize and prioritize the wide sharing within the care team of protocols for caring for newborns undergoing therapeutic hypothermia and their families to ensure consistency and comprehensive support.
- *Personalized Counseling*: Provide personalized counseling on the short- and long-term outcomes to parents before discharge, preparing them for the next steps and any challenges that may arise.

These recommendations aim to support not only the physical health of the newborn but also the emotional and psychological well-being of the family, which is integral to the overall care and recovery process and related outcomes.

**3.3. Future Directions**

***3.3.1. Mild hypoxic-ischemic encephalopathy Cases***

Therapeutic hypothermia is primarily reserved for moderate and severe cases of hypoxic-ischemic encephalopathy, largely based on historical data gathered before the clinical introduction of therapeutic hypothermia, which indicated no adverse outcomes in mild cases (111,112). For more details on the severity classification of neonatal hypoxic-ischemic encephalopathy and definitions used in the randomized clinical trials that informed these guidelines, please refer to Supplementary Table. Recent studies, however, are increasingly reporting detrimental effects observed on MRI scans in mild cases, affecting up to one out of five cases (113–116).

A significant limitation is the definitions and protocols used across different studies, the progressive nature of the disease, and the need for rapid assessment, compared to the more extensive but time-consuming protocols used in historical studies. For example, the original study that classified neonatal hypoxic-ischemic encephalopathy into three main categories - mild (with a favorable prognosis), moderate (with a favorable prognosis in about half of the cases), and severe (with an unfavorable prognosis) - was based on serial evaluations every 12 hours during the first 6 days of life and daily thereafter, supplemented with EEG and neuroimaging evidence (117). Such detailed assessments are now unfeasible when there is a critical need to rapidly assess the neurological status of these newborns to promptly initiate hypothermia in eligible cases. Moreover, neurological signs can evolve during the first 72 hours post-insult, thus a condition appearing mild within the first 6 hours (the threshold time to initiate hypothermia) might later manifest a significantly worse status and would have been classified as moderate or severe in historical studies (118,119).

***3.3.2. Preterm and low birth weight newborns***

There is currently no clear consensus regarding gestational age and birth weight thresholds for maintaining a positive benefit/risk ratio for therapeutic hypothermia. There is also significant heterogeneity in the thresholds used in existing literature, as summarized in Supplementary Table. Importantly, evaluating the neurological state of newborns becomes more challenging as gestational age decreases, due to the increasing difficulty in distinguishing signs and characteristics of hypoxia from those associated with prematurity.

***3.3.3. Combination with other neuroprotective strategies***

Several adjunctive strategies have been proposed to enhance neuroprotection in neonates susceptible to brain injury undergoing therapeutic hypothermia:

- *Melatonin*: Recent evidence suggests that melatonin may reduce cerebral damage and improve cognitive and neurological outcomes at 18 months for newborns with hypoxic-ischemic encephalopathy undergoing therapeutic hypothermia. However, these findings are based on studies involving small groups of patients, which may limit the generalizability of the results (44,120).
- *Erythropoietin*: A study involving 50 newborns undergoing therapeutic hypothermia demonstrated its efficacy in reducing detectable lesions on MRI scans when used at high intravenous dosages. Nonetheless, a subsequent randomized trial found no advantages in using erythropoietin in this neonatal population, and it was associated with a higher frequency of serious complications (121,122).
- *Topiramate*: The NeoNATI trial explored the combination of therapeutic hypothermia with topiramate but did not identify any significant improvement in outcomes (103). While topiramate may be considered a potential option for managing neonatal seizures, more evidence is needed to support its efficacy and safety in this context.
- *Magnesium Sulfate*: Recent studies have reported a favorable safety profile for the administration of magnesium sulfate in newborns undergoing therapeutic hypothermia. However, these studies did not detect any significant benefits regarding long-term outcomes (123,124).
- *Xenon*: The safety and efficacy of xenon were evaluated in the TOBY-Xe trial, which concluded that no clear advantages were detectable with its use (125).

In conclusion, while several alternative neuroprotective strategies have been investigated to complement therapeutic hypothermia in newborns affected by hypoxic-ischemic encephalopathy, no definitive recommendations can be made based on current evidence.

**Bibliography**

1. Brozek JL, Canelo-Aybar C, Akl EA, Bowen JM, Bucher J, Chiu WA, et al. GRADE Guidelines 30: the GRADE approach to assessing the certainty of modeled evidence-An overview in the context of health decision-making. J Clin Epidemiol. 2021 Jan;129:138–50.

2. Gluckman PD, Wyatt JS, Azzopardi D, Ballard R, Edwards AD, Ferriero DM, et al. Selective head cooling with mild systemic hypothermia after neonatal encephalopathy: multicentre randomised trial. Lancet. 2005 Feb 19;365(9460):663–70.

3. Shankaran S, Laptook AR, Ehrenkranz RA, Tyson JE, McDonald SA, Donovan EF, et al. Whole-body hypothermia for neonates with hypoxic-ischemic encephalopathy. N Engl J Med. 2005 Oct 13;353(15):1574–84.

4. Azzopardi DV, Strohm B, Edwards AD, Dyet L, Halliday HL, Juszczak E, et al. Moderate hypothermia to treat perinatal asphyxial encephalopathy. N Engl J Med. 2009 Oct 1;361(14):1349–58.

5. Simbruner G, Mittal RA, Rohlmann F, Muche R, neo.nEURO.network Trial Participants. Systemic hypothermia after neonatal encephalopathy: outcomes of neo.nEURO.network RCT. Pediatrics. 2010 Oct;126(4):e771-778.

6. Jacobs SE, Morley CJ, Inder TE, Stewart MJ, Smith KR, McNamara PJ, et al. Whole-body hypothermia for term and near-term newborns with hypoxic-ischemic encephalopathy: a randomized controlled trial. Arch Pediatr Adolesc Med. 2011 Aug;165(8):692–700.

7. Laptook AR, Shankaran S, Tyson JE, Munoz B, Bell EF, Goldberg RN, et al. Effect of Therapeutic Hypothermia Initiated After 6 Hours of Age on Death or Disability Among Newborns With Hypoxic-Ischemic Encephalopathy: A Randomized Clinical Trial. JAMA. 2017 Oct 24;318(16):1550–60.

8. Monnelly V, Becher JC. Sudden unexpected postnatal collapse. Early Hum Dev. 2018 Nov;126:28–31.

9. Brito S, Sampaio I, Dinis A, Proença E, Vilan A, Soares E, et al. Use of Therapeutic Hypothermia in Sudden Unexpected Postnatal Collapse: A Retrospective Study. Acta Med Port. 2021 June 1;34(6):442–50.

10. Pejovic NJ, Herlenius E. Unexpected collapse of healthy newborn infants: risk factors, supervision and hypothermia treatment. Acta Paediatr. 2013 July;102(7):680–8.

11. Cornet MC, Maton P, Langhendries JP, Marion W, Marguglio A, Smeets S, et al. [Use of therapeutic hypothermia in sudden unexpected postnatal collapse]. Arch Pediatr. 2014 Sept;21(9):1006–10.

12. Smit E, Liu X, Jary S, Cowan F, Thoresen M. Cooling neonates who do not fulfil the standard cooling criteria - short- and long-term outcomes. Acta Paediatr. 2015 Feb;104(2):138–45.

13. Filippi L, Laudani E, Tubili F, Calvani M, Bartolini I, Donzelli G. Incidence of Sudden Unexpected Postnatal Collapse in the Therapeutic Hypothermia Era. Am J Perinatol. 2017 June 1;34(13):1362–7.

14. Zhou W hao, Cheng G qiang, Shao X mei, Liu X zhi, Shan R bing, Zhuang D yi, et al. Selective head cooling with mild systemic hypothermia after neonatal hypoxic-ischemic encephalopathy: a multicenter randomized controlled trial in China. J Pediatr. 2010 Sept;157(3):367–72, 372.e1-3.

15. Jacobs SE, Berg M, Hunt R, Tarnow-Mordi WO, Inder TE, Davis PG. Cooling for newborns with hypoxic ischaemic encephalopathy. Cochrane Database Syst Rev. 2013 Jan 31;2013(1):CD003311.

16. Shah PS, Perlman M. Time courses of intrapartum asphyxia: neonatal characteristics and outcomes. Am J Perinatol. 2009 Jan;26(1):39–44.

17. Perlman JM. Summary proceedings from the neurology group on hypoxic-ischemic encephalopathy. Pediatrics. 2006 Mar;117(3 Pt 2):S28-33.

18. Azzopardi D. Clinical management of the baby with hypoxic ischaemic encephalopathy. Early Hum Dev. 2010 June;86(6):345–50.

19. Wyatt JS, Gluckman PD, Liu PY, Azzopardi D, Ballard R, Edwards AD, et al. Determinants of Outcomes After Head Cooling for Neonatal Encephalopathy. Pediatrics. 2007 May 1;119(5):912–21.

20. Polderman KH. Induced hypothermia and fever control for prevention and treatment of neurological injuries. Lancet. 2008 June 7;371(9628):1955–69.

21. Laptook A, Tyson J, Shankaran S, McDonald S, Ehrenkranz R, Fanaroff A, et al. Elevated temperature after hypoxic-ischemic encephalopathy: risk factor for adverse outcomes. Pediatrics. 2008 Sept;122(3):491–9.

22. Craig JV, Lancaster GA, Williamson PR, Smyth RL. Temperature measured at the axilla compared with rectum in children and young people: systematic review. BMJ. 2000 Apr 29;320(7243):1174–8.

23. Kendall GS, Kapetanakis A, Ratnavel N, Azzopardi D, Robertson NJ, Cooling on Retrieval Study Group. Passive cooling for initiation of therapeutic hypothermia in neonatal encephalopathy. Arch Dis Child Fetal Neonatal Ed. 2010 Nov;95(6):F408-412.

24. Fairchild K, Sokora D, Scott J, Zanelli S. Therapeutic hypothermia on neonatal transport: 4-year experience in a single NICU. J Perinatol. 2010 May;30(5):324–9.

25. Torre Monmany N, Behrsin J, Leslie A. Servo-controlled cooling during neonatal transport for babies with hypoxic-ischaemic encephalopathy is practical and beneficial: Experience from a large UK neonatal transport service. J Paediatr Child Health. 2019 May;55(5):518–22.

26. Landry MA, Doyle LW, Lee K, Jacobs SE. Axillary temperature measurement during hypothermia treatment for neonatal hypoxic-ischaemic encephalopathy. Arch Dis Child Fetal Neonatal Ed. 2013 Jan;98(1):F54-58.

27. Shalak LF, Laptook AR, Velaphi SC, Perlman JM. Amplitude-integrated electroencephalography coupled with an early neurologic examination enhances prediction of term infants at risk for persistent encephalopathy. Pediatrics. 2003 Feb;111(2):351–7.

28. Weeke LC, Vilan A, Toet MC, van Haastert IC, de Vries LS, Groenendaal F. A Comparison of the Thompson Encephalopathy Score and Amplitude-Integrated Electroencephalography in Infants with Perinatal Asphyxia and Therapeutic Hypothermia. Neonatology. 2017;112(1):24–9.

29. Parmentier CEJ, de Vries LS, Toet MC, van Haastert IC, Koopman C, Weeke LC, et al. Increased Use of Therapeutic Hypothermia in Infants with Milder Neonatal Encephalopathy due to Presumed Perinatal Asphyxia. Neonatology. 2020 Aug 19;117(4):488–94.

30. van Leuven K, Groenendaal F, Toet MC, Schobben AF a. M, Bos S a. J, de Vries LS, et al. Midazolam and amplitude-integrated EEG in asphyxiated full-term neonates. Acta Paediatr. 2004 Sept;93(9):1221–7.

31. Shany E, Benzaquen O, Friger M, Richardson J, Golan A. Influence of antiepileptic drugs on amplitude-integrated electroencephalography. Pediatr Neurol. 2008 Dec;39(6):387–91.

32. Deshpande P, Jain A, McNamara PJ. Effect of Phenobarbitone on Amplitude-Integrated Electroencephalography in Neonates with Hypoxic-Ischemic Encephalopathy during Hypothermia. Neonatology. 2020;117(6):721–8.

33. al Naqeeb N, Edwards AD, Cowan FM, Azzopardi D. Assessment of neonatal encephalopathy by amplitude-integrated electroencephalography. Pediatrics. 1999 June;103(6 Pt 1):1263–71.

34. Murray DM, Boylan GB, Ryan CA, Connolly S. Early EEG findings in hypoxic-ischemic encephalopathy predict outcomes at 2 years. Pediatrics. 2009 Sept;124(3):e459-467.

35. Briatore E, Ferrari F, Pomero G, Boghi A, Gozzoli L, Micciolo R, et al. EEG findings in cooled asphyxiated newborns and correlation with site and severity of brain damage. Brain Dev. 2013 May;35(5):420–6.

36. Lamblin MD, Walls Esquivel E, André M. The electroencephalogram of the full-term newborn: Review of normal features and hypoxic-ischemic encephalopathy patterns. Neurophysiologie Clinique/Clinical Neurophysiology. 2013 Dec 1;43(5):267–87.

37. Glass HC, Wusthoff CJ, Shellhaas RA, Tsuchida TN, Bonifacio SL, Cordeiro M, et al. Risk factors for EEG seizures in neonates treated with hypothermia: a multicenter cohort study. Neurology. 2014 Apr 8;82(14):1239–44.

38. Dilena R, Raviglione F, Cantalupo G, Cordelli DM, De Liso P, Di Capua M, et al. Consensus protocol for EEG and amplitude-integrated EEG assessment and monitoring in neonates. Clin Neurophysiol. 2021 Apr;132(4):886–903.

39. Hagmann CF, Robertson NJ, Azzopardi D. Artifacts on electroencephalograms may influence the amplitude-integrated EEG classification: a qualitative analysis in neonatal encephalopathy. Pediatrics. 2006 Dec;118(6):2552–4.

40. Marics G, Csekő A, Vásárhelyi B, Zakariás D, Schuster G, Szabó M. Prevalence and etiology of false normal aEEG recordings in neonatal hypoxic-ischaemic encephalopathy. BMC Pediatr. 2013 Nov 22;13:194.

41. Thoresen M. Patient selection and prognostication with hypothermia treatment. Semin Fetal Neonatal Med. 2010 Oct;15(5):247–52.

42. Kuratani J, Pearl PL, Sullivan L, Riel-Romero RMS, Cheek J, Stecker M, et al. American Clinical Neurophysiology Society Guideline 5: Minimum Technical Standards for Pediatric Electroencephalography. J Clin Neurophysiol. 2016 Aug;33(4):320–3.

43. Balduini W, Weiss MD, Carloni S, Rocchi M, Sura L, Rossignol C, et al. Melatonin pharmacokinetics and dose extrapolation after enteral infusion in neonates subjected to hypothermia. J Pineal Res. 2019 May;66(4):e12565.

44. Jerez-Calero A, Salvatierra-Cuenca MT, Benitez-Feliponi Á, Fernández-Marín CE, Narbona-López E, Uberos-Fernández J, et al. Hypothermia Plus Melatonin in Asphyctic Newborns: A Randomized-Controlled Pilot Study. Pediatr Crit Care Med. 2020 July;21(7):647–55.

45. MacLennan A. A template for defining a causal relation between acute intrapartum events and cerebral palsy: international consensus statement. BMJ. 1999 Oct 16;319(7216):1054–9.

46. Executive summary: Neonatal encephalopathy and neurologic outcome, second edition. Report of the American College of Obstetricians and Gynecologists’ Task Force on Neonatal Encephalopathy. Obstet Gynecol. 2014 Apr;123(4):896–901.

47. Overview | Intrapartum care | Guidance | NICE [Internet]. NICE; 2023 [cited 2025 Mar 4]. Available from: https://www.nice.org.uk/guidance/ng235

48. Westgate J, Garibaldi JM, Greene KR. Umbilical cord blood gas analysis at delivery: a time for quality data. Br J Obstet Gynaecol. 1994 Dec;101(12):1054–63.

49. Altanis S, Elrahman I. Sampling of umbilical cord blood gases after operative deliveries. J Obstet Gynaecol. 2007 Aug;27(6):571–3.

50. White CRH, Doherty DA, Kohan R, Newnham JP, Pennell CE. Evaluation of selection criteria for validating paired umbilical cord blood gas samples: an observational study. BJOG. 2012 June;119(7):857–65.

51. Lynn A, Beeby P. Cord and placenta arterial gas analysis: the accuracy of delayed sampling. Arch Dis Child Fetal Neonatal Ed. 2007 July;92(4):F281-285.

52. Battin MR, Dezoete JA, Gunn TR, Gluckman PD, Gunn AJ. Neurodevelopmental outcome of infants treated with head cooling and mild hypothermia after perinatal asphyxia. Pediatrics. 2001 Mar;107(3):480–4.

53. Vesoulis ZA, Liao SM, Rao R, Trivedi SB, Cahill AG, Mathur AM. Re-examining the Arterial Cord Blood Gas pH Screening Criteria in Neonatal Encephalopathy. Arch Dis Child Fetal Neonatal Ed. 2018 July;103(4):F377–82.

54. Malin GL, Morris RK, Khan KS. Strength of association between umbilical cord pH and perinatal and long term outcomes: systematic review and meta-analysis. BMJ. 2010 May 13;340:c1471.

55. Yeh P, Emary K, Impey L. The relationship between umbilical cord arterial pH and serious adverse neonatal outcome: analysis of 51,519 consecutive validated samples. BJOG. 2012 June;119(7):824–31.

56. Olofsson P. Umbilical cord pH, blood gases, and lactate at birth: normal values, interpretation, and clinical utility. Am J Obstet Gynecol. 2023 May;228(5S):S1222–40.

57. Yamada NK, Szyld E, Strand ML, Finan E, Illuzzi JL, Kamath-Rayne BD, et al. 2023 American Heart Association and American Academy of Pediatrics Focused Update on Neonatal Resuscitation: An Update to the American Heart Association Guidelines for Cardiopulmonary Resuscitation and Emergency Cardiovascular Care. Circulation. 2024 Jan 2;149(1):e157–66.

58. Wyckoff MH, Wyllie J, Aziz K, de Almeida MF, Fabres J, Fawke J, et al. Neonatal Life Support: 2020 International Consensus on Cardiopulmonary Resuscitation and Emergency Cardiovascular Care Science With Treatment Recommendations. Circulation. 2020 Oct 20;142(16_suppl_1):S185–221.

59. Lindacher V, Altebaeumer P, Marlow N, Matthaeus V, Straszewski IN, Thiele N, et al. European Standards of Care for Newborn Health-A project protocol. Acta Paediatr. 2021 May;110(5):1433–8.

60. Kendall AB, Scott PA, Karlsen KA. The S.T.A.B.L.E.® Program: the evidence behind the 2012 update. J Perinat Neonatal Nurs. 2012;26(2):147–57.

61. Thoresen M, Whitelaw A. Cardiovascular changes during mild therapeutic hypothermia and rewarming in infants with hypoxic-ischemic encephalopathy. Pediatrics. 2000 July;106(1 Pt 1):92–9.

62. Lista G, Castoldi F, Cavigioli F, Bianchi S, Fontana P, La Verde A. Ventilatory management of asphyxiated infant during hypothermia. J Matern Fetal Neonatal Med. 2011 Oct;24 Suppl 1:67–8.

63. Pappas A, Shankaran S, Laptook AR, Langer JC, Bara R, Ehrenkranz RA, et al. Hypocarbia and adverse outcome in neonatal hypoxic-ischemic encephalopathy. J Pediatr. 2011 May;158(5):752-758.e1.

64. Lopez Laporte MA, Wang H, Sanon PN, Barbosa Vargas S, Maluorni J, Rampakakis E, et al. Association between hypocapnia and ventilation during the first days of life and brain injury in asphyxiated newborns treated with hypothermia. J Matern Fetal Neonatal Med. 2019 Apr;32(8):1312–20.

65. Szakmar E, Jermendy A, El-Dib M. Respiratory management during therapeutic hypothermia for hypoxic-ischemic encephalopathy. J Perinatol. 2019 June;39(6):763–73.

66. Dawson JA, Kamlin COF, Vento M, Wong C, Cole TJ, Donath SM, et al. Defining the reference range for oxygen saturation for infants after birth. Pediatrics. 2010 June;125(6):e1340-1347.

67. Sabir H, Jary S, Tooley J, Liu X, Thoresen M. Increased inspired oxygen in the first hours of life is associated with adverse outcome in newborns treated for perinatal asphyxia with therapeutic hypothermia. J Pediatr. 2012 Sept;161(3):409–16.

68. Giannakis S, Ruhfus M, Markus M, Stein A, Hoehn T, Felderhoff-Mueser U, et al. Mechanical Ventilation, Partial Pressure of Carbon Dioxide, Increased Fraction of Inspired Oxygen and the Increased Risk for Adverse Short-Term Outcomes in Cooled Asphyxiated Newborns. Children (Basel). 2021 May 21;8(6):430.

69. Vento M, Escrig R, Saenz P, Izquierdo I, Sastre J, Viña J. Does Oxygen Concentration Used for Resuscitation Influence Outcome of Asphyxiated Newly Born Infants Treated With Hypothermia? Pediatrics. 2006 June 1;117(6):2326–8.

70. Soar J, Maconochie I, Wyckoff MH, Olasveengen TM, Singletary EM, Greif R, et al. 2019 International Consensus on Cardiopulmonary Resuscitation and Emergency Cardiovascular Care Science With Treatment Recommendations: Summary From the Basic Life Support; Advanced Life Support; Pediatric Life Support; Neonatal Life Support; Education, Implementation, and Teams; and First Aid Task Forces. Circulation. 2019 Dec 10;140(24):e826–80.

71. Lingappan K, Kaiser JR, Srinivasan C, Gunn AJ. Relationship between PCO2 and unfavorable outcome in infants with moderate-to-severe hypoxic ischemic encephalopathy. Pediatr Res. 2016 Aug;80(2):204–8.

72. Laffey JG, Kavanagh BP. Hypocapnia. N Engl J Med. 2002 July 4;347(1):43–53.

73. Curley G, Laffey JG, Kavanagh BP. Bench-to-bedside review: carbon dioxide. Crit Care. 2010;14(2):220.

74. Lasso Pirot A, Fritz KI, Ashraf QM, Mishra OP, Delivoria-Papadopoulos M. Effects of severe hypocapnia on expression of bax and bcl-2 proteins, DNA fragmentation, and membrane peroxidation products in cerebral cortical mitochondria of newborn piglets. Neonatology. 2007;91(1):20–7.

75. Klinger G, Beyene J, Shah P, Perlman M. Do hyperoxaemia and hypocapnia add to the risk of brain injury after intrapartum asphyxia? Arch Dis Child Fetal Neonatal Ed. 2005 Jan;90(1):F49-52.

76. Hallberg B, Olson L, Bartocci M, Edqvist I, Blennow M. Passive induction of hypothermia during transport of asphyxiated infants: a risk of excessive cooling. Acta Paediatr. 2009 June;98(6):942–6.

77. Hagan JL. Meta-analysis comparing temperature on arrival at the referral hospital of newborns with hypoxic ischemic encephalopathy cooled with a servo-controlled device versus no device during transport. J Neonatal Perinatal Med. 2021;14(1):29–41.

78. Akula VP, Joe P, Thusu K, Davis AS, Tamaresis JS, Kim S, et al. A randomized clinical trial of therapeutic hypothermia mode during transport for neonatal encephalopathy. J Pediatr. 2015 Apr;166(4):856-861.e1-2.

79. Goel N, Mohinuddin SM, Ratnavel N, Kempley S, Sinha A. Comparison of Passive and Servo-Controlled Active Cooling for Infants with Hypoxic-Ischemic Encephalopathy during Neonatal Transfers. Am J Perinatol. 2017 Jan;34(1):19–25.

80. Stafford TD, Hagan JL, Sitler CG, Fernandes CJ, Kaiser JR. Therapeutic Hypothermia During Neonatal Transport: Active Cooling Helps Reach the Target. Ther Hypothermia Temp Manag. 2017 June;7(2):88–94.

81. Leon RL, Krause KE, Sides RS, Koch MB, Trautman MS, Mietzsch U. Therapeutic Hypothermia in Transport Permits Earlier Treatment Regardless of Transfer Distance. Am J Perinatol. 2022 Apr;39(6):633–9.

82. Arriagada S, Huang H, Fletcher K, Giannone P. Prevention of excessive hypothermia in infants with hypoxic ischemic encephalopathy prior to admission to a quaternary care center: a neonatal outreach educational project. J Perinatol. 2019 Oct;39(10):1417–27.

83. Lago P, Spada C, Lugli L, Garetti E, Pirelli A, Savant Levet P, et al. Pain management during therapeutic hypothermia in newborn infants with hypoxic-ischaemic encephalopathy. Acta Paediatr. 2020 Mar;109(3):628–9.

84. Ancora G, Lago P, Garetti E, Merazzi D, Savant Levet P, Bellieni CV, et al. Evidence-based clinical guidelines on analgesia and sedation in newborn infants undergoing assisted ventilation and endotracheal intubation. Acta Paediatr. 2019 Feb;108(2):208–17.

85. Strohm B, Hobson A, Brocklehurst P, Edwards AD, Azzopardi D, UK TOBY Cooling Register. Subcutaneous fat necrosis after moderate therapeutic hypothermia in neonates. Pediatrics. 2011 Aug;128(2):e450-452.

86. Saygı Aİ, Özdamar Ö, Gün İ, Emirkadı H, Müngen E, Akpak YK. Comparison of maternal and fetal outcomes among patients undergoing cesarean section under general and spinal anesthesia: a randomized clinical trial. Sao Paulo Med J. 2015;133(3):227–34.

87. Cocchi E, Pini R, Gallipoli A, Stella M, Antonazzo P, Marchetti F, et al. Impact of general vs. neuraxial anesthesia on neonatal outcomes in non-elective cesarean sections. Front Pediatr. 2025 Mar 3;13:1518456.

88. Joshi M, Muneer J, Mbuagbaw L, Goswami I. Analgesia and sedation strategies in neonates undergoing whole-body therapeutic hypothermia: A scoping review. PLoS One. 2023;18(12):e0291170.

89. Bäcke P, Bruschettini M, Sibrecht G, Thernström Blomqvist Y, Olsson E. Pharmacological interventions for pain and sedation management in newborn infants undergoing therapeutic hypothermia. Cochrane Database Syst Rev. 2022 Nov 10;11(11):CD015023.

90. Natarajan G, Shankaran S, Laptook AR, McDonald SA, Pappas A, Hintz SR, et al. Association between sedation-analgesia and neurodevelopment outcomes in neonatal hypoxic-ischemic encephalopathy. J Perinatol. 2018 Aug;38(8):1060–7.

91. Róka A, Melinda KT, Vásárhelyi B, Machay T, Azzopardi D, Szabó M. Elevated morphine concentrations in neonates treated with morphine and prolonged hypothermia for hypoxic ischemic encephalopathy. Pediatrics. 2008 Apr;121(4):e844-849.

92. Favié LMA, Groenendaal F, van den Broek MPH, Rademaker CMA, de Haan TR, van Straaten HLM, et al. Pharmacokinetics of morphine in encephalopathic neonates treated with therapeutic hypothermia. PLoS One. 2019;14(2):e0211910.

93. Acun C, Ali M, Liu W, Karnati S, Fink K, Aly H. Effectiveness and Safety of Dexmedetomidine in Neonates With Hypoxic Ischemic Encephalopathy Undergoing Therapeutic Hypothermia. J Pediatr Pharmacol Ther. 2024 June 10;29(3):232.

94. Naveed M, Bondi DS, Shah PA. Dexmedetomidine Versus Fentanyl for Neonates With Hypoxic Ischemic Encephalopathy Undergoing Therapeutic Hypothermia. J Pediatr Pharmacol Ther. 2022;27(4):352–7.

95. Elliott M, Burnsed J, Heinan K, Letzkus L, Andris R, Fairchild K, et al. Effect of dexmedetomidine on heart rate in neonates with hypoxic ischemic encephalopathy undergoing therapeutic hypothermia. J Neonatal Perinatal Med. 2022;15(1):47–54.

96. Elliott M, Fairchild K, Zanelli S, McPherson C, Vesoulis Z. Dexmedetomidine During Therapeutic Hypothermia: A Multicenter Quality Initiative. Hospital Pediatrics. 2023 Dec 20;14(1):30–6.

97. Cosnahan AS, Angert RM, Jano E, Wachtel EV. Dexmedetomidine versus intermittent morphine for sedation of neonates with encephalopathy undergoing therapeutic hypothermia. J Perinatol. 2021 Sept;41(9):2284–91.

98. Surkov D. Using of dexmedetomidine in term neonates with hypoxic-ischemic encephalopathy. Medicni perspektivi. 2019 Nov 6;24(2):24–33.

99. O’Mara K, Weiss MD. Dexmedetomidine for Sedation of Neonates with HIE Undergoing Therapeutic Hypothermia: A Single-Center Experience. AJP Rep. 2018 July;8(3):e168–73.

100. Cocchi E, Shabani J, Aceti A, Ancora G, Corvaglia L, Marchetti F. Dexmedetomidine as a Promising Neuroprotective Sedoanalgesic in Neonatal Therapeutic Hypothermia: A Systematic Review and Meta-Analysis. Neonatology. 2025 May 2;122(4):495–504.

101. Pressler RM, Abend NS, Auvin S, Boylan G, Brigo F, Cilio MR, et al. Treatment of seizures in the neonate: Guidelines and consensus-based recommendations—Special report from the ILAE Task Force on Neonatal Seizures. Epilepsia. 2023 Oct;64(10):2550–70.

102. Booth D, Evans DJ. Anticonvulsants for neonates with seizures. Cochrane Database Syst Rev. 2004 Oct 18;(4):CD004218.

103. Filippi L, Fiorini P, Catarzi S, Berti E, Padrini L, Landucci E, et al. Safety and efficacy of topiramate in neonates with hypoxic ischemic encephalopathy treated with hypothermia (NeoNATI): a feasibility study. J Matern Fetal Neonatal Med. 2018 Apr;31(8):973–80.

104. Geiger I, Kostenzer J, Matthäus V, On Behalf Of The Escnh Chair Committee null, Mader S, Zimmermann LJI. Reassessing and Extending the European Standards of Care for Newborn Health: How to Keep Reference Standards in Line with Current Evidence. Children (Basel). 2024 Feb 1;11(2):179.

105. Hall ES, Greenberg JM. Estimating community-level costs of preterm birth. Public Health. 2016 Dec;141:222–8.

106. Lopez-Maestro M, De la Cruz J, Perapoch-Lopez J, Gimeno-Navarro A, Vazquez-Roman S, Alonso-Diaz C, et al. Eight principles for newborn care in neonatal units: Findings from a national survey. Acta Paediatrica. 2020;109(7):1361–8.

107. Heringhaus A, Blom MD, Wigert H. Becoming a parent to a child with birth asphyxia-From a traumatic delivery to living with the experience at home. Int J Qual Stud Health Well-being. 2013 Apr 30;8:1–13.

108. Craig AK, James C, Bainter J, Evans S, Gerwin R. Parental perceptions of neonatal therapeutic hypothermia; emotional and healing experiences. J Matern Fetal Neonatal Med. 2020 Sept;33(17):2889–96.

109. Craig AK, Gerwin R, Bainter J, Evans S, James C. Exploring Parent Experience of Communication About Therapeutic Hypothermia in the Neonatal Intensive Care Unit. Adv Neonatal Care. 2018 Apr;18(2):136–43.

110. Bell A, Rubin L, Davis J, Golding J, FMCOG OA, Carter C. The birth experience and subsequent maternal caregiving attitudes and behavior: A birth cohort study. Arch Womens Ment Health. 2019 Oct;22(5):613–20.

111. Robertson C, Finer N. Term infants with hypoxic-ischemic encephalopathy: outcome at 3.5 years. Dev Med Child Neurol. 1985 Aug;27(4):473–84.

112. Robertson CM, Finer NN, Grace MG. School performance of survivors of neonatal encephalopathy associated with birth asphyxia at term. J Pediatr. 1989 May;114(5):753–60.

113. Conway JM, Walsh BH, Boylan GB, Murray DM. Mild hypoxic ischaemic encephalopathy and long term neurodevelopmental outcome - A systematic review. Early Hum Dev. 2018 May;120:80–7.

114. Chalak LF, Nguyen KA, Prempunpong C, Heyne R, Thayyil S, Shankaran S, et al. Prospective research in infants with mild encephalopathy identified in the first six hours of life: neurodevelopmental outcomes at 18-22 months. Pediatr Res. 2018 Dec;84(6):861–8.

115. Reiss J, Sinha M, Gold J, Bykowski J, Lawrence SM. Outcomes of Infants with Mild Hypoxic Ischemic Encephalopathy Who Did Not Receive Therapeutic Hypothermia. Biomed Hub. 2019;4(3):1–9.

116. Finder M, Boylan GB, Twomey D, Ahearne C, Murray DM, Hallberg B. Two-Year Neurodevelopmental Outcomes After Mild Hypoxic Ischemic Encephalopathy in the Era of Therapeutic Hypothermia. JAMA Pediatr. 2020 Jan 1;174(1):48–55.

117. Sarnat HB, Sarnat MS. Neonatal encephalopathy following fetal distress. A clinical and electroencephalographic study. Arch Neurol. 1976 Oct;33(10):696–705.

118. Boylan GB, Kharoshankaya L, Mathieson SR. Diagnosis of seizures and encephalopathy using conventional EEG and amplitude integrated EEG. Handb Clin Neurol. 2019;162:363–400.

119. Lugli L, Guidotti I, Pugliese M, Roversi MF, Bedetti L, Della Casa Muttini E, et al. Polygraphic EEG Can Identify Asphyxiated Infants for Therapeutic Hypothermia and Predict Neurodevelopmental Outcomes. Children (Basel). 2022 Aug 9;9(8):1194.

120. Aly H, Elmahdy H, El-Dib M, Rowisha M, Awny M, El-Gohary T, et al. Melatonin use for neuroprotection in perinatal asphyxia: a randomized controlled pilot study. J Perinatol. 2015 Mar;35(3):186–91.

121. Wu YW, Mathur AM, Chang T, McKinstry RC, Mulkey SB, Mayock DE, et al. High-Dose Erythropoietin and Hypothermia for Hypoxic-Ischemic Encephalopathy: A Phase II Trial. Pediatrics. 2016 June;137(6):e20160191.

122. Wu YW, Comstock BA, Gonzalez FF, Mayock DE, Goodman AM, Maitre NL, et al. Trial of Erythropoietin for Hypoxic–Ischemic Encephalopathy in Newborns. New England Journal of Medicine. 2022 July 13;387(2):148–59.

123. Gulczynska E, Gadzinowski J, Nowiczewski M, Sobolewska B, Caputa J, Maczko A, et al. Feasibility and Safety of Combining Therapeutic Hypothermia with Magnesium Sulfate Administration, in the Management of Neonates with Hypoxic Ischemic Encephalopathy - Randomized Control Trial. In: Neonatal and Pediatric Medicine [Internet]. 2018 [cited 2025 Jan 12]. Available from: https://www.omicsonline.org/open-access/feasibility-and-safety-of-combining-therapeutic-hypothermia-with-magnesium-sulfate-administration-in-the-management-of-neonates-wi-2572-4983-1000165-104409.html

124. Kumar C, Adhisivam B, Bobby Z, Bhat BV. Magnesium Sulfate as an Adjunct to Therapeutic Hypothermia in the Management of Term Infants with Hypoxic-Ischemic Encephalopathy: A Randomized, Parallel-Group, Controlled Trial. Indian J Pediatr. 2023 Sept;90(9):886–92.

125. Azzopardi D, Chew AT, Deierl A, Huertas A, Robertson NJ, Tusor N, et al. Prospective qualification of early cerebral biomarkers in a randomised trial of treatment with xenon combined with moderate hypothermia after birth asphyxia. EBioMedicine. 2019 Sept;47:484–91.
