# Supplementary material for: Therapeutic hypothermia in newborns: evidence-based guidelines from a systematic review
Source: Ital J Pediatr. 2026 Apr 27;52:103. doi: 10.1186/s13052-026-02266-x (PMC13255436; doi:10.1186/s13052-026-02266-x)
Supplement: Supplementary file 2 — Supplementary material 2 [file 13052_2026_2266_MOESM2_ESM.pdf]

MEDLINE (OVID)

#	results num.	research question
1	676152	Infant, Newborn/
2	62361	Infant, Premature/
3	8719	Infant, Small for Gestational Age/
4	20371	Infant, Low Birth Weight/
5	10025	Infant, Very Low Birth Weight/
6	395	Infant, Postmature/
7	11	Infant, Large for Gestational Age/
8	39635	Infant, Newborn, Diseases/
9	3250	Term Birth/
10	1908783	(Newborn or Neonat* or Preterm* or premature or "Low birth weight" or lbw or elbw or vlbw or "Low birth weights" or "Low birthweight" or "Low birthweights" or "pre-terms" or "Pre-term" or "Small gestational age" or SGA or "Extremely premature" or "Postmature" or "Large gestational age" or LGA or term).ab,ti.
11	2284196	1 or 2 or 3 or 4 or 5 or 6 or 7 or 8 or 9 or 10
12	15130	Hypothermia/
13	22074	Induced Hypothermia/
14	22074	Therapeutic Hypothermia/
15	22074	Hypothermia, Therapeutic/
16	119959	("hypothermia" or "therapeutic hypothermia" or "cooling" or "freezing").ab,ti.
17	130936	12 or 13 or 14 or 15 or 16
18	4739	Neurodevelopmental Disorders/
19	50601	Child Development/
20	22477	Developmental Disabilities/
21	14694	Learning Disorders/
22	2900	Communication Disorders/
23	3252	Motor Skills Disorders/
24	60328	Intellectual Disability/
25	25989	Autistic Disorder/
26	244912	("neurodevelopment" or "neurodevelop*" or "brain development" or "neurological development" or "cognitive development" or "neurocognitive development" or "developmental delay*" or "psychomotor development" or "developmental milestone*" or "learning disability" or "learning disorder*" or "communication disorder*" or "speech disorder*" or "autism spectrum disorder*" or "autistic disorder*" or "motor skill disorder*" or "motor delay*" or "intellectual disability" or "intellectual impairment" or "cognitive impairment" or "developmental disability" or "developmental coordination disorder" Or "global developmental delay" or "cognitive deficit*" or "cognitive dysfunction").ab,ti.
27	374132	18 or 19 or 20 or 21 or 22 or 23 or 24 or 25 or 26
28	545	11 and 17 and 27
29	54	28 and "Randomized Controlled Trial".sa_pubt.
30	10	28 and "Clinical Trial".sa_pubt.
31	312234	("RCT" or "randomized clinical trial" or "clinical trial" or "randomized controlled trial").ab,ti.
32	38	28 and 31
58	73	29 or 30 or 31

Scopus

#	results num.	research question
1	2660263	(newborn:ab,ti,kw OR neonat*:ab,ti,kw OR preterm*:ab,ti,kw OR premature:ab,ti,kw OR 'low birth weight':ab,ti,kw OR lbw:ab,ti,kw OR elbw:ab,ti,kw OR vlbw:ab,ti,kw OR 'low birth weights':ab,ti,kw OR 'low birthweight':ab,ti,kw OR 'low birthweights':ab,ti,kw OR 'pre-terms':ab,ti,kw OR 'pre-term':ab,ti,kw OR 'small gestational age':ab,ti,kw OR sga:ab,ti,kw OR 'extremely premature':ab,ti,kw OR 'postmature':ab,ti,kw OR 'large gestational age':ab,ti,kw OR lga:ab,ti,kw OR term:ab,ti,kw)
2	146366	('hypothermia':ab,ti,kw OR 'therapeutic hypothermia':ab,ti,kw OR 'cooling':ab,ti,kw OR 'freezing':ab,ti,kw)
3	374698	('neurodevelopment':ab,ti,kw OR 'neurodevelop*':ab,ti,kw OR 'brain development':ab,ti,kw OR 'neurological development':ab,ti,kw OR 'cognitive development':ab,ti,kw OR 'neurocognitive development':ab,ti,kw OR 'developmental delay*':ab,ti,kw OR 'psychomotor development':ab,ti,kw OR 'developmental milestone*':ab,ti,kw OR 'learning disability':ab,ti,kw OR 'learning disorder*':ab,ti,kw OR 'communication disorder*':ab,ti,kw OR 'speech disorder*':ab,ti,kw OR 'autism spectrum disorder*':ab,ti,kw OR 'autistic disorder*':ab,ti,kw OR 'motor skill disorder*':ab,ti,kw OR 'motor delay*':ab,ti,kw OR 'intellectual disability':ab,ti,kw OR 'intellectual impairment':ab,ti,kw OR 'cognitive impairment':ab,ti,kw OR 'developmental disability':ab,ti,kw OR 'developmental coordination disorder':ab,ti,kw OR 'global developmental delay':ab,ti,kw OR 'cognitive deficit*':ab,ti,kw OR 'cognitive dysfunction':ab,ti,kw)
4	490342	('rct':ab,ti,kw OR 'randomized clinical trial':ab,ti,kw OR 'clinical trial':ab,ti,kw OR 'randomized controlled trial':ab,ti,kw)
5	206	#1 and #2 and #3 and #4

Embase

#	results num.	research question
1	2660263	(newborn:ab,ti,kw OR neonat*:ab,ti,kw OR preterm*:ab,ti,kw OR premature:ab,ti,kw OR 'low birth weight':ab,ti,kw OR lbw:ab,ti,kw OR elbw:ab,ti,kw OR vlbw:ab,ti,kw OR 'low birth weights':ab,ti,kw OR 'low birthweight':ab,ti,kw OR 'low birthweights':ab,ti,kw OR 'pre-terms':ab,ti,kw OR 'pre-term':ab,ti,kw OR 'small gestational age':ab,ti,kw OR sga:ab,ti,kw OR 'extremely premature':ab,ti,kw OR 'postmature':ab,ti,kw OR 'large gestational age':ab,ti,kw OR lga:ab,ti,kw OR term:ab,ti,kw)
2	146366	('hypothermia':ab,ti,kw OR 'therapeutic hypothermia':ab,ti,kw OR 'cooling':ab,ti,kw OR 'freezing':ab,ti,kw)
3	374698	('neurodevelopment':ab,ti,kw OR 'neurodevelop*':ab,ti,kw OR 'brain development':ab,ti,kw OR 'neurological development':ab,ti,kw OR 'cognitive development':ab,ti,kw OR 'neurocognitive development':ab,ti,kw OR 'developmental delay*':ab,ti,kw OR 'psychomotor development':ab,ti,kw OR 'developmental milestone*':ab,ti,kw OR 'learning disability':ab,ti,kw OR 'learning disorder*':ab,ti,kw OR 'communication disorder*':ab,ti,kw OR 'speech disorder*':ab,ti,kw OR 'autism spectrum disorder*':ab,ti,kw OR 'autistic disorder*':ab,ti,kw OR 'motor skill disorder*':ab,ti,kw OR 'motor delay*':ab,ti,kw OR 'intellectual disability':ab,ti,kw OR 'intellectual impairment':ab,ti,kw OR 'cognitive impairment':ab,ti,kw OR 'developmental disability':ab,ti,kw OR 'developmental coordination disorder':ab,ti,kw OR 'global developmental delay':ab,ti,kw OR 'cognitive deficit*':ab,ti,kw OR 'cognitive dysfunction':ab,ti,kw)
4	490342	('rct':ab,ti,kw OR 'randomized clinical trial':ab,ti,kw OR 'clinical trial':ab,ti,kw OR 'randomized controlled trial':ab,ti,kw)
5	33	#1 and #2 and #3 and #4

Cochrane

#	results num.	research question
1	22692	MeSH descriptor: [Infant, Newborn] explode all trees
2	5419	MeSH descriptor: [Infant, Premature] explode all trees
3	401	MeSH descriptor: [Infant, Small for Gestational Age] explode all trees
4	2873	MeSH descriptor: [Infant, Low Birth Weight] explode all trees
5	1313	MeSH descriptor: [Infant, Very Low Birth Weight] explode all trees
6	10	MeSH descriptor: [Infant, Postmature] explode all trees
7	0	MeSH descriptor: [Infant, Large for Gestational Age] explode all trees
8	9169	MeSH descriptor: [Infant, Newborn, Diseases] explode all trees
9	206	MeSH descriptor: [Term Birth] explode all trees
10	249558	Newborn or Neonat* or Preterm* or Premature or "Low birth weight" or lbw or elbw or vlbw or "Low birth weights" or "Low birthweight" or "Low birthweights" or "pre-terms" or "Pre-term" or "Small gestational age" or SGA or "Extremely premature" or "Postmature" or "Large gestational age" or LGA or term
11	251938	#1 or #2 or #3 or #4 or #5 or #6 or #7 or #8 or #9 or #10
12	918	MeSH descriptor: [Hypothermia] explode all trees
13	1252	MeSH descriptor: [Hypothermia, Induced] explode all trees
14	8676	"hypothermia" or "therapeutic hypothermia" or "cooling" or "freezing"
15	8676	#12 or #13 or #14
16	10768	MeSH descriptor: [Neurodevelopmental Disorders] explode all trees
17	3346	MeSH descriptor: [Child Development] explode all trees
18	797	MeSH descriptor: [Developmental Disabilities] explode all trees
19	774	MeSH descriptor: [Learning Disabilities] explode all trees
20	2461	MeSH descriptor: [Communication Disorders] explode all trees
21	245	MeSH descriptor: [Motor Skills Disorders] explode all trees
22	1954	MeSH descriptor: [Intellectual Disability] explode all trees
23	1520	MeSH descriptor: [Autistic Disorder] explode all trees
24	26989	"neurodevelopment" or "neurodevelopmental disorder" or "brain development" or "neurological development" or "cognitive development" or "neurocognitive development" or "developmental delay" or "psychomotor development" or "developmental milestone" or "learning disability" or "learning disorder" or "communication disorder" or "speech disorder" or "autism spectrum disorder" or "autistic disorder" or "motor skill disorder" or "motor delay" or "intellectual disability" or "intellectual impairment" or "cognitive impairment" or "developmental disability" or "developmental coordination disorder" or "global developmental delay" or "cognitive deficit" or "cognitive dysfunction"
25	26989	#16 or #17 or #18 or #19 or #20 or #21 or #22 or #23 or #24
26	820127	"RCT" or "randomized clinical trial" or "clinical trial" or "randomized controlled trial"
27	55	#11 and #15 and #25 and #26

Web of Science

#	results num.	research question
1	5225269	TS=(Newborn or Neonat* or Preterm* or premature or "Low birth weight" or lbw or elbw or vlbw or "Low birth weights" or "Low birthweight" or "Low birthweights" or "pre-terms" or "Pre-term" or "Small gestational age" or SGA or "Extremely premature" or "Postmature" or "Large gestational age" or LGA or term)
2	475033	TS=("hypothermia" or "therapeutic hypothermia" or "cooling" or "freezing")
3	358993	TS=("neurodevelopment" or "neurodevelop*" or "brain development" or "neurological development" or "cognitive development" or "neurocognitive development" or "developmental delay*" or "psychomotor development" or "developmental milestone*" or "learning disability" or "learning disorder*" or "communication disorder*" or "speech disorder*" or "autism spectrum disorder*" or "autistic disorder*" or "motor skill disorder*" or "motor delay*" or "intellectual disability" or "intellectual impairment" or "cognitive impairment" or "developmental disability" or "developmental coordination disorder" or "global developmental Delay" or "cognitive deficit*" or "cognitive dysfunction")
4	463538	TS=("RCT" or "randomized clinical trial" or "clinical trial" Or "randomized controlled trial")
5	35	#1 and #2 and #3 and #4
